# An improved organotypic cell culture system to study tissue-resident macrophages *ex vivo*

**DOI:** 10.1016/j.crmeth.2022.100260

**Published:** 2022-07-26

**Authors:** Philipp Aktories, Philippe Petry, Paulo Glatz, Geoffroy Andrieux, Alexander Oschwald, Hannah Botterer, Oliver Gorka, Daniel Erny, Melanie Boerries, Philipp Henneke, Olaf Groß, Marco Prinz, Katrin Kierdorf

**Affiliations:** 1 Institute of Neuropathology, Faculty of Medicine, University of Freiburg, 79106 Freiburg, Germany; 2 Faculty of Biology, University of Freiburg, 79104 Freiburg, Germany; 3 Institute of Medical Bioinformatics and Systems Medicine, Medical Center, Faculty of Medicine, University of Freiburg, 79106 Freiburg, Germany; 4 German Cancer Consortium (DKTK), Partner Site Freiburg, and German Cancer Research Center (DKFZ), 69120 Heidelberg, Germany; 5 Berta-Ottenstein-Program for Advanced Clinician Scientists, Faculty of Medicine, University of Freiburg, 79106 Freiburg, Germany; 6 CIBSS-Center for Integrative Biological Signaling Studies, University of Freiburg, 79104 Freiburg, Germany; 7 Institute for Immunodeficiency, Center for Chronic Immunodeficiency, and Center for Pediatrics and Adolescent Medicine, Medical Center, Faculty of Medicine, University of Freiburg, 79106 Freiburg, Germany; 8 Center for Basics in NeuroModulation (NeuroModulBasics), Faculty of Medicine, University of Freiburg, 79106 Freiburg, Germany; 9 Signaling Research Centers BIOSS and CIBSS, University of Freiburg, 79104 Freiburg, Germany

**Keywords:** macrophages, organotypic cell culture, tissue normoxia, Kupffer cells, microglia, alveolar macrophages, peritoneal macrophages, bone marrow-derived macrophages, tissue-resident macrophages

## Abstract

Tissue-resident macrophages (TRMs) perform organ-specific functions that are dependent on factors such as hematopoietic origin, local environment, and biological influences. A diverse range of *in vitro* culture systems have been developed to decipher TRM functions, including bone marrow-derived macrophages (BMDMs), induced pluripotent stem cell (iPSC)-derived TRMs, or immortalized cell lines. However, despite the usefulness of such systems, there are notable limitations. Attempts to culture primary macrophages often require purification of cells and lack a high cell yield and consistent phenotype. Here, we aimed to address these limitations by establishing an organotypic primary cell culture protocol. We obtained long-term monocultures of macrophages derived from distinct organs without prior purification using specific growth factors and tissue normoxic conditions that largely conserved a TRM-like identity *in vitro*. Thus, this organotypic system offers an ideal screening platform for primary macrophages from different organs that can be used for a wide range of assays and readouts.

## Highlights

• A modified cell culture protocol allows long-term culture of TRM-like cells
• Protocols have been developed for brain, liver, peritoneum, and lung
• TRM-like cells maintain core features of their TRM counterparts *in vivo*
• TRM-like cells can be used for various functional assays *in vitro*

## Motivation

Efficient cell culture systems, especially for primary TRMs, are rare, and most of them have limitations such as low cell yield or artificial cell activation. In this study, we aimed to address several of these limitations and establish an organotypic cell culture protocol that can be easily established in many labs and applied to many different TRMs. With this cell culture approach, we would like to provide a reliable source for immunologists to study primary TRMs from different organs *in vitro.*

## Graphical abstract

**Figure:**
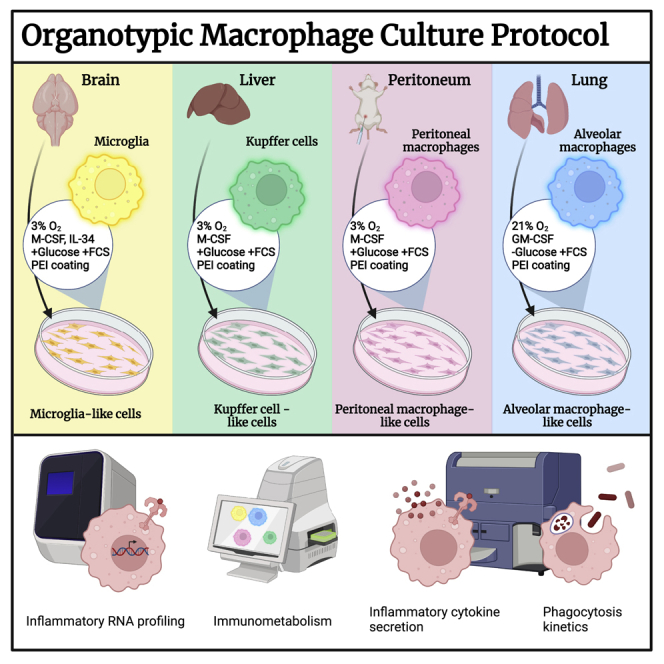

## Teaser

*In vitro* cultures are essential to study tissue-resident macrophages (TRMs) but are hampered by low cell yields or other limitations. To provide reliable TRM sources *in vitro*, Aktories et al. established cell culture protocols for TRM-like cells from brain, liver, peritoneum, and lung and subsequently employed TRM-like cells for functional assays.

## Introduction

Tissue-resident macrophages (TRMs) are modulated by the specific environment of their host tissue, such as Kupffer cells (KCs) in the liver, microglia (MG) in the brain, peritoneal macrophages (PMs) in the peritoneum, or alveolar macrophages (AMs) in the lung (Guilliams et al., 2020). Most adult TRMs are long lived and derived from embryonic progenitors (Ginhoux et al., 2010; Gomez Perdiguero et al., 2015; Guilliams et al., 2013; Hoeffel et al., 2015; Schulz et al., 2012), with some exemptions, such as TRMs in oral mucosa or intestine (Bain et al., 2014; Capucha et al., 2015). TRMs serve specialized functions to maintain tissue homeostasis all while poised to rapidly transition to an inflammatory phenotype upon injury or infection (Kierdorf and Dionne, 2016). Subsequently, TRMs initiate tissue repair processes to restore tissue homeostasis (Wynn and Vannella, 2016). Chronic inflammatory pathologies are characterized by a detrimental contribution of macrophages (Voet et al., 2019). Given that the identity and tissue specificity of TRMs are imprinted by the tissue environment (Bonnardel et al., 2019; Hagemeyer et al., 2016; Kolter et al., 2020; Schulz et al., 2012), their steady-state and activation phenotypes can be radically different. Thus, investigating these biologically relevant nuances continues to be a topic of interest within the field of immunology.

Cell culture protocols have been developed to explore TRM function. Thus far, many studies have relied on bone marrow-derived macrophages (BMDMs), which do not per se represent TRM heterogeneity (Bailey et al., 2020; Stanley and Heard, 1977). Other approaches include oncogene-transformed cell lines (Blasi et al., 1990; Kreuzburg-Duffy and Macdonald, 1991), which allow an easy maintenance and a high cell yield, but often with an activated phenotype (Bocchini et al., 1992; Napoli et al., 2009). During the past decade, induced pluripotent stem cells (iPSCs) and iPSC-derived organoids presented a novel opportunity to generate and study TRMs *in vitro* (Lee et al., 2018; Takata et al., 2017), although these challenging techniques require a high level of expertise and financial resources. Available *in vitro* protocols for primary TRMs rely on complex dissociation (e.g., enzymatic digestion) and purification steps (e.g., fluorescence activated cell sorting [FACS] or magnetic-activated cell sorting [MACS]). These steps can result in cell activation, as recently demonstrated for MG of the CNS (Haimon et al., 2018; Mattei et al., 2020). Further, even optimized culture protocols result in low cell yields, and functional assays on sorted cells are limited and restricted to a few hours or days post isolation. In light of these limiting factors, we aimed to develop a more organotypic, high-yield, cost-efficient, and long-term monoculture protocol for TRMs of different tissues.

Here, we established a primary organotypic cell culture for TRMs without prior cell purification. “TRM-like” cells (TRM-LCs) can be sustained for weeks, show negligible contamination with other cell types, and largely share gene expression profiles and surface markers with their *in vivo* counterparts. As a result, the generated TRM-LCs offer efficient readouts in functional and metabolic assays. Thus, TRM-LCs offer immunologists an optimized *in vitro* platform to study the functions of the body’s various TRM populations.

## Results

### Combination of adapted culture conditions allows *in vitro* maintenance of pure TRM-LCs from distinct organs

To overcome the above-mentioned obstacles for TRM cultures, we adapted three major culture conditions: (1) pre-coating with polyethylenimine (PEI) to facilitate a high cell yield; (2) cell culture media supplemented with specific growth factors and only one partial medium change to preserve host tissue-derived trophic factors; and (3) organotypic oxygen levels to avoid activation and metabolic alterations.

**Figure 1:**
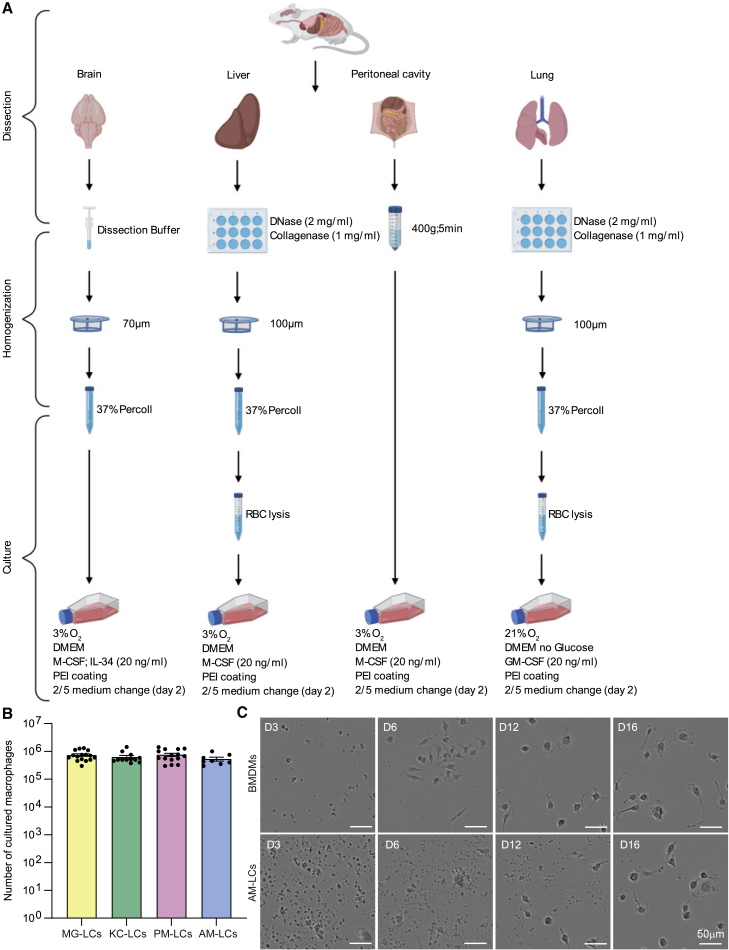
TRM-LCs are efficiently maintained in culture by a combination of supportive culture coating, specific medium supplementation, and tissue normoxic oxygen levels (A) Isolation protocol for TRM-LCs from brain, liver, peritoneum, and lung. RBC, red blood cell. (B) Absolute cell numbers of MG-LCs (yellow), KC-LCs (green), PM-LCs (pink), and AM-LC (blue) after 2 weeks in culture. Mean ± SEM is shown; cells per culture flask are shown, n = 8–17/group. (C) Bright-field images of BMDMs (upper row) and AM-LCs (lower row) are shown from day (D) 3, 6, 12, and 16 in the Incucyte system. One of two independent experiments is shown. Scale bars, 50 μm. See also Figure S1 and Videos S1 and S2.

We generated MG-like cells (MG-LCs), KC-like cells (KC-LCs), AM-like cells (AM-LCs), and PM-like cells (PM-LCs) by plating single-cell suspensions of their respective organs (Figure 1A; STAR Methods). To verify the cellular composition of the obtained cell pellets for culture (Figure S1), we found that the main myeloid populations included in the cell pellets were TRMs, except for the cell pellet of the liver, where granulocytes were more abundant (Figure S1B). Cells from each organ were plated in PEI-coated tissue culture flasks (Figure 1A), where the PEI coating effectively eliminated contaminating host cells and supported monocultures of TRM-LCs within 14 days.

The medium was supplemented with growth factors in a tissue-specific manner: macrophage colony-stimulating factor (M-CSF) for KC- and PM-LCs, M-CSF and interleukin (IL)-34 for MG-LCs, and granulocyte-macrophage colony-stimulating factor (GM-CSF) for AM-LCs (Figure 1A) (Greter et al., 2012; Guilliams et al., 2013; Kana et al., 2019; Yoshida et al., 1990). To account for other organ-derived trophic factors, a single partial medium change (2/5) was performed 2 days after plating to preserve these factors in the medium (Figure 1A). Furthermore, KC-, PM-, and MG-LCs were supplemented with high glucose (4.5 g/L), while AM-LCs were glucose deprived to reflect the low glucose availability in the lung alveoli (Woods et al., 2020) (Figure 1A). Atmospheric oxygen levels constitute a hyperoxic condition for most cells in culture (Carreau et al., 2011; Jagannathan et al., 2016). In the brain, liver, and peritoneal cavity, physiological oxygen levels are between 3% and 6%, whereas lung alveoli contain approximately 14.5% (Carreau et al., 2011; Jagannathan et al., 2016). Consequently, we adapted the oxygen levels of our cultures to replicate the concentrations *in vivo*. KC-, PM-, and MG-LCs were cultured at 3% oxygen, while AM-LCs were cultivated at atmospheric oxygen levels (Figure 1A). After 14 days, cell cultures of each macrophage population were obtained, with population sizes ranging between 300,000 and 1,500,000 cells per flask (Figure 1B).

To follow the culture development, we imaged AM-LCs compared with BMDMs over the course of 2 weeks after the partial medium change with an Incucyte system (Figure 1C). As the Incucyte system must be utilized at atmospheric oxygen levels, AM-LCs were imaged here. BMDMs reached confluence after 7 days, with all cells acquiring characteristic macrophage morphology (Figure 1C; Video S1). In contrast, AM-LCs took much longer to develop into densely populated cultures. After an initially low amount of cell seedings and a high abundance of cell debris, cells that resembled macrophages appeared a few days post plating and proliferated in clusters (Figure 1C; Video S2). After 16 days, AM-LCs were seen with typical macrophage morphology and the cells did not expand further.

Video S1. Time lapse of cultured BMDMs over 2 weeks in the Incucyte system, related to Figure 1 Bright-field images were taken every 2 h. One of two independent experiments is shown. Scale bar, 400 μm.

Video S2. Time lapse of cultured AM-LCs over 2 weeks in the Incucyte system, related to Figure 1 Bright-field images were taken every 2 h. One of two independent experiments is shown. Scale bar, 400 μm.

**Figure 2:**
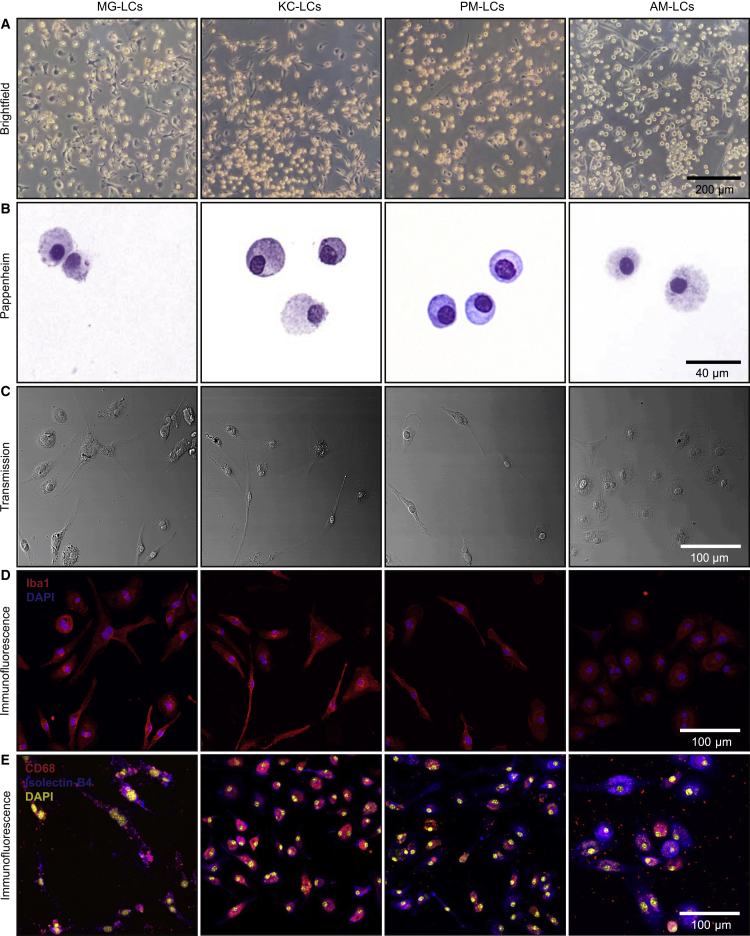
TRM-LCs develop a typical cell morphology and macrophage marker expression after 2 weeks in defined culture conditions (A) Bright-field images of TRM-like cell cultures after 2 weeks. One of three independent experiments is shown. Scale bar, 200 μm. (B) Pappenheim stainings of cytospins from TRM-LCs. Scale bar, 40 μm. (C) Confocal transmission light images of TRM-LCs. One of three independent experiments is shown. Scale bar, 100 μm. (D) Confocal immunofluorescence images of TRM-LCs. Iba1 is shown in red and DAPI is shown in blue. One of three independent experiments is shown. Scale bar, 100 μm. (E) Confocal immunofluorescence images of TRM-LCs after 2 weeks. CD68 is shown in red, isolectin B4 is shown in blue, and DAPI is shown in yellow. One independent experiment is shown. Scale bar, 100 μm.

After 2 weeks, the organotypic system produced densely populated TRM-LCs from all organs of interest. TRM-LCs developed with round, bipolar, but also ramified, morphologies, all of which are typical of TRMs *in vitro* (Figure 2A). Pappenheim staining of TRM-LC cytospins showed light purple staining of mononuclear myeloid cells without distinct structures of heterochromatin or signs of polymorphic nuclei, excluding a contamination with neutrophils or eosinophils (Figure 2B). Transmission light imaging of TRM-LCs with a confocal microscope once again showcased typical bipolar/ramified macrophage morphologies with phagocytic inclusions (Figure 2C). Immunostaining for ionized calcium-binding adapter molecule 1 (Iba1), expressed by many macrophages, except AMs (Köhler, 2007), revealed pure cultures of Iba1^+^ cells at day 14. Of note, AM-LCs upregulated Iba1 *in vitro* (Figure 2D). In addition, TRM-LCs showed abundant expression of the lysosomal marker CD68, broadly expressed in TRMs, as well as surface binding of isolectin B4, described for myeloid cells (Figure 2E). Hence, we confirmed that our approach allows us to obtain densely populated macrophage cultures from an organ of interest, without the need for purification by cell sorting.

### Cultured TRM-LCs maintain core macrophage surface markers of respective TRM populations *ex vivo* with some alterations

**Figure 3:**
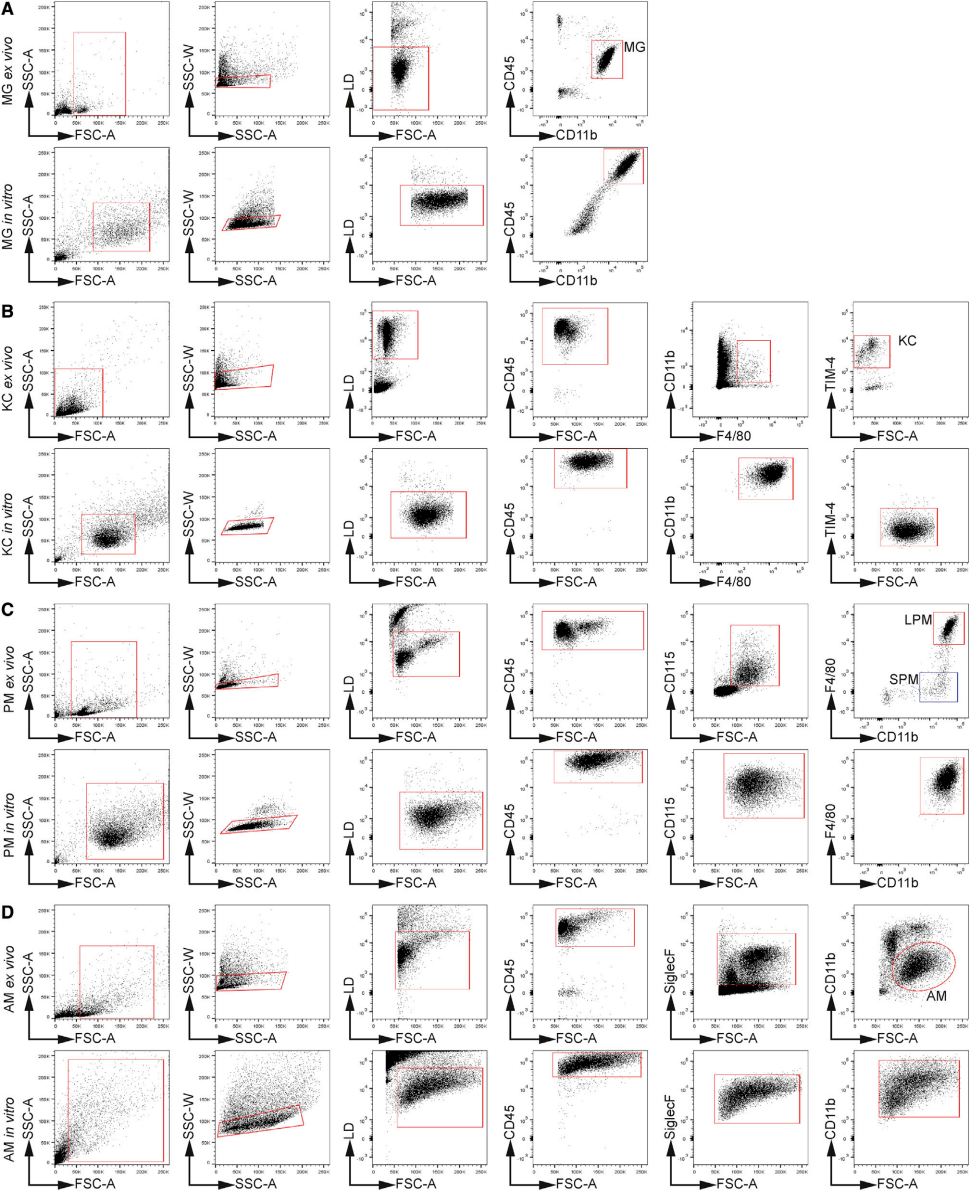
TRM-LCs can be identified *in vitro* by organ-specific FACS gating strategies Gating strategies are shown from left to right; gates are indicated in red. One of three independent experiments is shown. Gating strategies are shown for *ex vivo* isolated MG (upper row) and *in vitro* cultured MG-LCs (lower row) (A); for *ex vivo* isolated KCs (upper row) and *in vitro* cultured KC-LCs (lower row) (B); for *ex vivo* isolated PMs (upper row), with blue gate indicating SPMs, and *in vitro* cultured PM-LCs (lower row) (C); and for *ex vivo* isolated AMs (upper row) and *in vitro* cultured AM-LCs (lower row) (D). See also Figure S2.

As described before, the isolation and subsequent *in vitro* culturing of TRMs often result in the loss of identifying surface markers, due to either the lack of host tissue cells or artificial activation. We compared cell-surface markers of purified MG, KCs, PMs, and AMs (“*ex vivo*”) with cultured TRM-LCs 14 days post plating (“*in vitro*”). Freshly isolated MG were identified as CD45^low^CD11b^+^ cells (Figure 3A), whereas cultured MG-LCs were CD45^high^CD11b^high^. MG-LCs also tended to be larger and had a higher granularity and autofluorescence, likely due to phagocytosed material *in vitro*. KCs were identified as CD45^+^CD11b^+^F4/80^+^TIM-4^+^ cells; but KC-LCs lost TIM-4 *in vitro* (Figure 3B). In the peritoneal lavage, PMs can be divided into large PMs (LPMs) (CD45^+^CD115^+^CD11b^+^F4/80^high^) and small PMs (SPMs) (CD45^+^CD115^+^CD11b^+^F4/80^low^) (Figure 3C); however, our culture system produced a pure macrophage population of CD45^+^CD115^+^CD11b^+^F4/80^high^ macrophages, suggesting that the system preferentially harbors LPM-LCs (Figure 3C). Freshly isolated AMs were identified as CD45^+^SiglecF^+^CD11b^low^ (Figure 3D) but showed higher CD11b expression and lower SiglecF expression *in vitro* (Figure 3D). TRM-LCs maintain several surface markers even after an extended culturing period, but with some alterations.

Next, we examined classical core macrophage surface markers and macrophage activation markers to exclude artificial activation *in vitro*. In cultured MG-LCs, we found a slight increase in the surface levels of the glycoprotein F4/80 compared with the *ex vivo* isolated MG, whereas other TRM-LCs showed consistent F4/80 levels compared with freshly isolated cells (Figure S2A). CD115 or colony-stimulating factor 1 receptor (Csf1r) is expressed on cultured and freshly isolated MG, KCs, and PMs, but was lost in AM-LCs (Figure S2B). Interestingly, major histocompatibility complex class II (MHC-II) is not expressed by MG-LCs *in vitro* and MG directly isolated from CNS. On the other hand, KC-, PM-, and AM-LCs still expressed MHC-II but at reduced levels compared with their *ex vivo* isolated counterparts (Figure S2C). Integrin subunit αx (Itgax or CD11 c) expression was low in adult MG from brain tissue and undetectable in cultured MG-LCs (Figure S2D), while *ex vivo* expression of CD11c was maintained in the other TRM-LCs. Notably, CD11c was more highly expressed on PM-LCs *in vitro* than on freshly isolated PMs (Figure S2D). Sialic acid-binding Ig-like lectin F (SiglecF) was highly expressed on freshly isolated AMs (Figure S2E). This AM-specific expression was preserved in culture, albeit to a reduced level (Figure S2E). T cell membrane protein 4 (TIM-4) was highly expressed on PM and KC populations *ex vivo* (Figure S2F), but both PM- and KC-LCs *in vitro* appeared to lose TIM-4 (Figure S2F). To verify that the surface-marker profile was not dictated by the culture conditions only, but rather maintained from the host tissue, we analyzed expression for F4/80, CD115, MHC-II, CD11c, SiglecF, and TIM-4 on BMDMs cultured under the distinct TRM-LC culture conditions (Figure S2G). We could not identify a culture-condition-specific signature on BMDMs resembling the surface expression of specific TRM-LCs (Figure S2G). Therefore, TRM-like macrophage populations seem to be not activated as a result of the culture system, despite the alteration of certain surface markers between the cultured TRM-LCs and their *in vivo* counterparts.

### TRM-LCs share a core transcriptomic signature with their TRM counterparts but with adaptations induced *in vitro*

**Figure 4:**
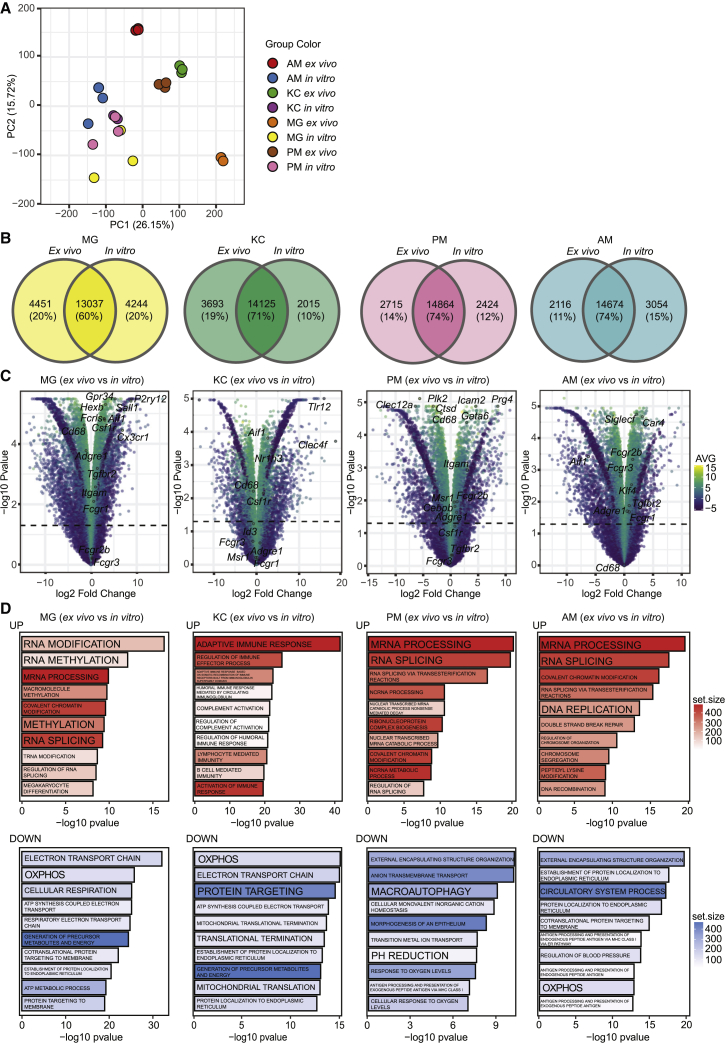
Transcriptomic profiling of TRM-LCs *in vitro* and freshly isolated TRMs *ex vivo* revealed culture-induced changes but maintenance of a core transcriptomic profile (A) Principal-component analysis (PCA) plot of analyzed FACS-sorted TRMs (“*ex vivo*”) and TRM-LCs (“*in vitro*”). Principle component 1 versus 2 are shown. Each dot represents one sample analyzed. Color code is shown in the key. (B) Venn diagrams depicting numbers of differentially regulated genes and shared genes between *ex vivo* isolated TRMs and their corresponding TRM-LCs *in vitro*. The overlap indicates genes shared between both groups (i.e., nonregulated genes). Genes were considered to be differentially regulated with a significant fold change of >1.5 and adjusted p < 0.05. (C) Volcano plots of differentially expressed genes between *ex vivo* isolated TRMs and their corresponding TRM-LCs *in vitro*. Typical TRM genes are highlighted. Color code represents the average log2 CPM value. (D) Gene ontology (GO) biological process term enrichment analysis in differentially expressed genes (*ex vivo* versus *in vitro*). Upper row shows the top 10 GO terms expressed significantly higher in the respective TRM population *ex vivo* compared with *in vitro*. The bottom row shows the top 10 GO terms expressed significantly lower in the respective TRM population *ex vivo* compared with *in vitro*. Gene set size is indicated by color scale. See also Table S1 and Figure S3.

Next we performed transcriptomic profiling of the TRM-LCs and compared them with their respective *in vivo* counterparts. Analyzed samples clustered separately according to their groups with only minor variations in a principle-component analysis (PCA); however, we saw that TRM-LCs (“*in vitro*”) and sorted TRMs (“*ex vivo*”) clustered apart (Figure 4A). We compared the number of shared and differentially expressed genes between TRM-LCs *in vitro* and their *in vivo* counterparts (Figure 4B; Table S1). For MG-LCs we found that 60% of the genes were still shared with FACS-sorted MG (Figure 4B). Similarly, we detected between 71% and 74% of genes to be shared between KC-, PM-, and also AM-LCs with their corresponding TRM population. When we compared expression levels of differentially expressed genes in *ex vivo* isolated TRMs with TRM-LCs, we found that several signature genes were indeed expressed higher in freshly isolated TRMs, but the specific tissue signature was not lost in TRM-like cultures (Figure 4C). For example, when we compared sorted MG with MG-LCs, we found that signature genes such as *P2ry12* or *Sall1* showed a high fold change, whereas others were regulated only to lower levels, such as *Fcrls*, *Gpr34*, or *Hexb* (Table S1). In line with this, the KC signature gene *Clec4f* was expressed higher in FACS-sorted KCs compared with cultured KC-LCs, whereas *Id3* remained unaltered (Table S1). Even though PMs expressed significantly higher levels of *Cebpb* and *Gata6* compared with PM-LCs, the detected fold change was only small between the sample groups (Table S1). Similarly, freshly isolated AMs expressed significantly higher levels of *Car4* and *Siglecf* compared with AM-LCs *in vitro*, but with a low fold change between both groups, indicating that these signature genes were still expressed (Table S1). Of note, we also confirmed SiglecF protein expression on the surface of AM-LCs (Figure 3D). Further comparing TRM-LCs with their corresponding TRM population *in vivo*, we defined differentially regulated gene ontology (GO) terms associated with RNA splicing and mRNA processing within the top 10 GO terms, which were expressed higher in freshly isolated TRM populations (Figure 4D). In contrast to other TRMs, freshly isolated KCs expressed higher levels of several GO terms associated with immune regulation compared with the KC-LCs (Figure 4D). Overall, we did not find other differentially regulated GO terms associated with macrophage function or macrophage differentiation in the top 10. We next compared shared regulated genes between all TRM-LCs to elucidate if there is a potential “core *in vitro* macrophage signature.” We identified only 254 induced genes and 275 downregulated genes in *ex vivo* TRMs compared with TRM-LCs, indicating no common core signature (Figure S3A). GO terms associated with chemotaxis and motility were expressed lower in *ex vivo* isolated TRMs compared with TRM-LCs, whereas GO terms associated with epigenetic modulations were downregulated (Figure S3B). Given that, we could henceforth prove that core transcriptomic profiles of TRM identities seem to be maintained in our organotypic system.

To confirm TRM signature gene expression *in vitro*, we additionally performed semi-quantitative gene expression analyses via qPCR for several signature genes. Here, eight genes were selected to exemplify TRM identity: *Hexb* and *Fcrls* for MG, *Id3* and *Nr1h3* for KCs, *Tgfb2* and *Cebpb* for PMs, and *Car4* and *Siglecf* for AMs.

As expected, MG-LCs expressed *HexB* and *Fcrls* at elevated levels compared with all other TRM-like cultures (Figure S3C), KC-LCs expressed *Id3* and *Nr1h3* at significantly higher levels compared with other cultured populations (Figure S3D), PM-LCs expressed *Tgfb2* at a significantly higher level compared with other TRM-LCs but not significantly elevated levels of *Cebpb* (Figure S3E), and AM-LCs showed a significantly higher expression of *Car4* and *Siglecf* compared with the other TRM-LCs (Figure S3F). To exclude that these signature genes were not artificially induced by the different culture conditions of the TRM-LCs, we cultured BMDMs in the respective culture conditions and analyzed again signature gene expression (Figures S3G–S3J). Here, we could not detect the induction of specific TRM gene signatures in BMDMs through the applied culture conditions (Figures S4G–S4J), reinforcing that the gene expression profiles were rather a result of their host tissue origin. Hence, TRM-LCs can serve as a powerful surrogate system but do not completely conserve TRM identity.

### TRM-like cultures do not express a monocyte-associated gene profile and are not directly derived from Ly6C^high^ monocytes

**Figure 5:**
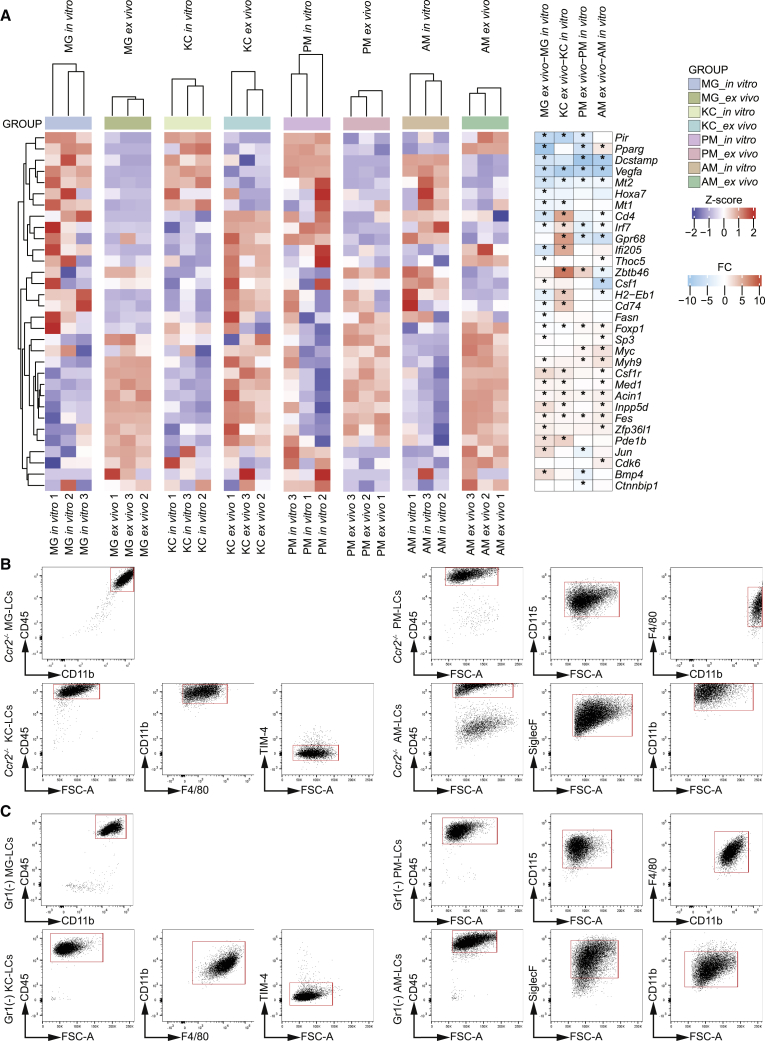
Cultured TRM-LCs do not show a monocyte differentiation profile and are not directly derived from Ly6C^high^ monocytes (A) Heatmap of monocyte-associated genes in TRMs *ex vivo* and TRM-LCs *in vitro*. *Z* score is shown for all samples *in vitro* and *ex vivo* (left). Color code for gene expression level is indicated in the key. Heatmap reflecting the fold change (FC) between distinct TRM-LCs *in vitro* and their *ex vivo* counterparts (right). Color code for gene expression level is indicated in the key. Asterisks indicate significant regulation (adjusted p < 0.05) from limma analysis. (B) Flow cytometry gating for TRM-LCs of *Ccr2*^−/−^ mice after 14 days *in vitro*. Gating strategies are shown from left to right, gates are indicated in red. Doublets and dead cells were excluded before. (C) Flow cytometry gating for TRM-LCs after MACS depletion of Gr1^+^ cells after 14 days *in vitro*. Gating strategies are shown from left to right, gates are indicated in red. Doublets and dead cells were excluded before. One of two independent experiments is shown. See also Figures S4 and S5.

Alterations in gene expression and protein levels for some signature genes could be explained by monocytes, which might give rise to TRM-LCs in the organotypic culture system. Hence, we investigated if monocytes could be the source of TRM-LCs *in vitro*. First, we looked into the transcriptomic data of the TRM populations *ex vivo* and TRM-LCs *in vitro* and compared the expression levels of genes associated with monocyte differentiation (GO: 0030224) and regulation of monocyte differentiation (GO: 0045655, 0045656, and 0045657) (Figure 5A). We did not observe an upregulation of these genes *in vitro* compared with the freshly isolated TRM populations. The genes were heterogeneously expressed across all TRM populations and TRM-LCs. We even found higher expression of genes associated with monocyte differentiation in *ex vivo* isolated KCs compared with the cultured KC-LCs (Figure 5A). Hence, we could not detect an upregulation of monocyte differentiation-associated genes in the organotypic cultures.

To further exclude Ly6C^high^ monocytes as a source of TRM-LCs, we used *Ccr2*^−/−^ mice, which have a reduced number of Ly6C^high^ monocytes in the blood (Serbina and Pamer, 2006), to generate TRM-LCs (Figure S4). After 2 weeks, we identified TRM-LCs from all organs occupying our cultures (Figure 5B), indicating that Ly6C^high^ monocytes are likely not a source of TRM-LCs. To confirm these results, we again created TRM-LCs from wild-type (WT) mice but depleted Ly6C^high^ monocytes and granulocytes via anti-Gr1 MACS from the cell pellets prior to plating. After efficient depletion of Gr1^+^ myeloid cells from the cell pellets (Figure S5), we again found normal establishment and abundance of TRM-LCs in the cultures from all four organs (Figure 5C). Thus far, we can exclude that the TRM-LCs in our organotypic cultures were monocyte-derived macrophages, but rather were descended from TRMs themselves or so-far-undescribed endogenous progenitors in the respective tissues.

### TRM-like populations show distinct phagocytic rates *in vitro*

**Figure 6:**
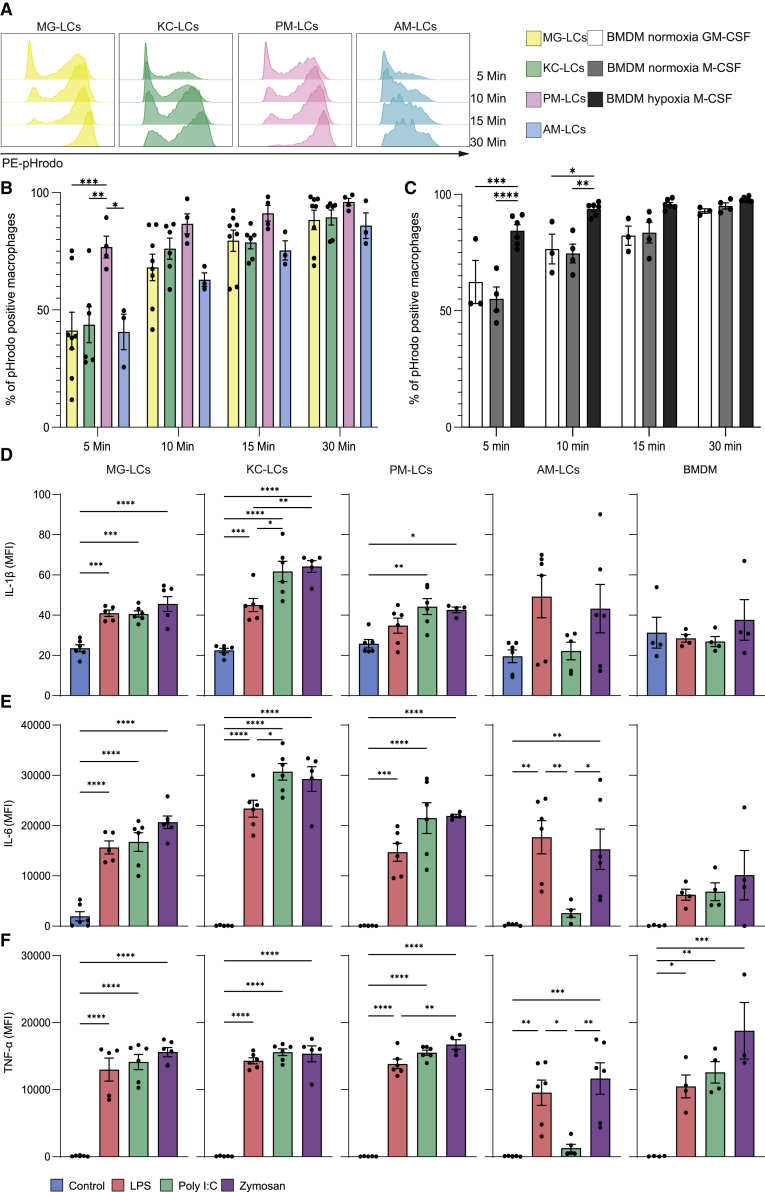
Tissue-resident macrophages show distinct phagocytic speeds and inflammatory gene expression upon stimulation *in vitro* MG-LCs are shown in yellow, KC-LCs in green, PM-LCs in pink, and AM-LCs in blue. One of three to eight independent experiments is shown. Cells were gated as depicted in Figure S7. (A) Representative histograms of PE-pHrodo labeling after 5, 10, 15, and 30 min of TRM-LCs phagocytosis of PE-pHrodo-coupled zymosan beads *in vitro*. (B) Quantification of percentage of TRM-LCs labeled with PE-pHrodo after 5, 10, 15, and 30 min. Mean ± SEM is shown; n = 3–8; ∗p < 0.05, ∗∗p < 0.01, and ∗∗∗p < 0.001. (C) Quantification of percentage of differently cultured BMDMs labeled with PE-pHrodo after 5, 10, 15, and 30 min. BMDMs in normoxia + GM-CSF are shown in white, BMDMs in normoxia + M-CSF are shown in gray, BMDMs in hypoxia + M-CSF are shown in dark gray. Mean ± SEM is shown; n = 3–6; ∗p < 0.05, ∗∗p < 0.01, and ∗∗∗p < 0.001. (D–F) Levels of secreted cytokines are measured in mean fluorescence intensity (MFI). Control is shown in blue, LPS in red, poly(I:C) in green, and zymosan in violet. Released cytokine levels of IL-1β are shown in (D), IL-6 in (E), and TNF-α in (F). Mean ± SEM is shown; n = 3/group; ∗p < 0.05, ∗∗p < 0.01, and ∗∗∗p < 0.001. See also Figures S6 and S7.

To explore the potential usage of TRM-LCs in different functional assays *in vitro*, we first analyzed the phagocytosis dynamics of TRM-LCs by using pHrodo zymosan-coupled beads. TRM-LCs were identified as CD45^+^CD11b^+^; cytochalasin D was used as a negative control (Figure S6). As anticipated, all TRM-LCs rapidly phagocytosed the beads, with each population demonstrating an increased number of pHrodo^+^ cells, up to over 80% after 30 min (Figures 6A and 6B). TRM-LCs from different organs showed profound differences in phagocytosis speed. After 5 min, PM-LCs showed a significantly higher percentage of pHrodo^+^ cells compared with the other TRM-LCs (Figure 6B). At 10 min, MG- and KC-LCs began to approach the higher percentage of pHrodo^+^ cells found in PM-LCs, whereas the phagocytic capacity of AM-LCs appeared to increase more gradually (Figure 6B). At 15 and 30 min, all TRM-LCs possessed near-equivalent loads of phagocytosed beads (Figure 6B). Our results suggest that PM-LCs are much faster at phagocytosing zymosan-coupled beads compared with the other TRM-LCs, whereas AM-LCs reacted more slowly to the stimulus. Of note, BMDMs cultured under the same conditions as PM- and KC-LCs behaved similar to PM-LCs but not KC-LCs, supporting our hypothesis that the function is not induced by the culture conditions directly (Figure 6C). Therefore, we demonstrated the potential use of TRM-LCs to test the phagocytic capabilities of different TRM-LCs.

### Inflammatory cytokine expression and release by TRM-LCs upon immune stimulation

Extending the functional analysis further, we evaluated cytokine expression and release by TRM-LCs upon treatment with different immune stimuli (Figures 6D–6F and S7). We treated TRM-LCs and BMDMs (M-CSF, 21% O_2_) with lipopolysaccharides (LPS), poly(I:C), and zymosan and analyzed the gene expression of *C-C motif chemokine ligand 2* (*Ccl2*), *Interleukin-1*β (*Il1b*), *Interleukin-6* (*Il6*), and *tumor necrosis factor alpha* (*Tnfa*) after 24 h. KC-LCs expressed significantly more *Il1b* after poly(I:C) stimulation compared with control, whereas the other TRM-LCs did not (Figure S7A). After LPS treatment, MG-LCs showed a significant induction of *Il1b*, and similarly, BMDMs significantly upregulated *Il1b* upon LPS stimulation, whereas PM- and AM-LCs did not (Figure S7A). *Il6* was heterogeneously induced in the TRM-LCs and BMDMs. Poly(I:C) and zymosan significantly induced *Il6* in MG-LCs compared with control (Figure S7B). Upon treatment with LPS, KC-LCs showed a significant upregulation of *Il6* compared with untreated KC-LCs (Figure S7B). Even though all TRM-LCs showed elevated levels of *Ccl2* in all treatment paradigms, none of these treatments induced a significant upregulation of *Ccl2* (Figure S7C). Expression of *Tnfa* upon LPS treatment was significantly elevated in MG- and KC-LCs (Figure S7D). However, AM-LCs, PM-LCs, and BMDMs showed only slightly elevated *Tnfa* levels (Figure S7D). Poly(I:C) and zymosan treatment did not significantly increase the expression of *Tnfa* in all TRM-like cultures analyzed (Figure S7D). The gene expression analysis after immune stimulation revealed a high heterogeneity between the different TRM-LCs. Furthermore, the distinct cytokine expression pattern in response to immunogenic stimuli by BMDMs compared with TRM-LCs reinforces the idea that BMDMs are not an ideal surrogate to mimic TRM behavior *in vitro*.

Subsequently, we aimed to analyze the release of cytokines into the cell culture supernatant 24 h after stimulation. We used LEGENDPlex bead-based assays to quantify the amount of secreted IL-1β, IL-6, and TNF-α via flow cytometry (Figures 6D–6F). As expected, in the absence of an inflammasome stimulus, we detected only very low levels of IL-1β secretion in TRM-LCs, most likely background signals due to IL-1β released by potential cell death (Figure 6D). In contrast, MG-, KC-, and PM-LCs significantly induced the release of IL-6 and TNF-α compared with untreated cells (Figures 6E and 6F). AM-LCs showed a significant release of IL-6 after LPS and zymosan treatment, but not after poly(I:C) treatment (Figure 6E). Of note, BMDMs did not show a significant release of IL-6 24 h after immune stimulation (Figure 6E) but released TNF-α (Figure 6F). Hence, TRM-LCs showed a distinct cytokine expression and release pattern to BMDMs after immune stimulation. Moreover, the cytokine induction varied between TRM-LCs from distinct host tissues. We can conclude that TRM-LCs could represent a cell culture tool to study cytokine release and inflammatory gene expression.

### Immunometabolic analysis of TRM-like cultures upon immune stimulation

**Figure 7:**
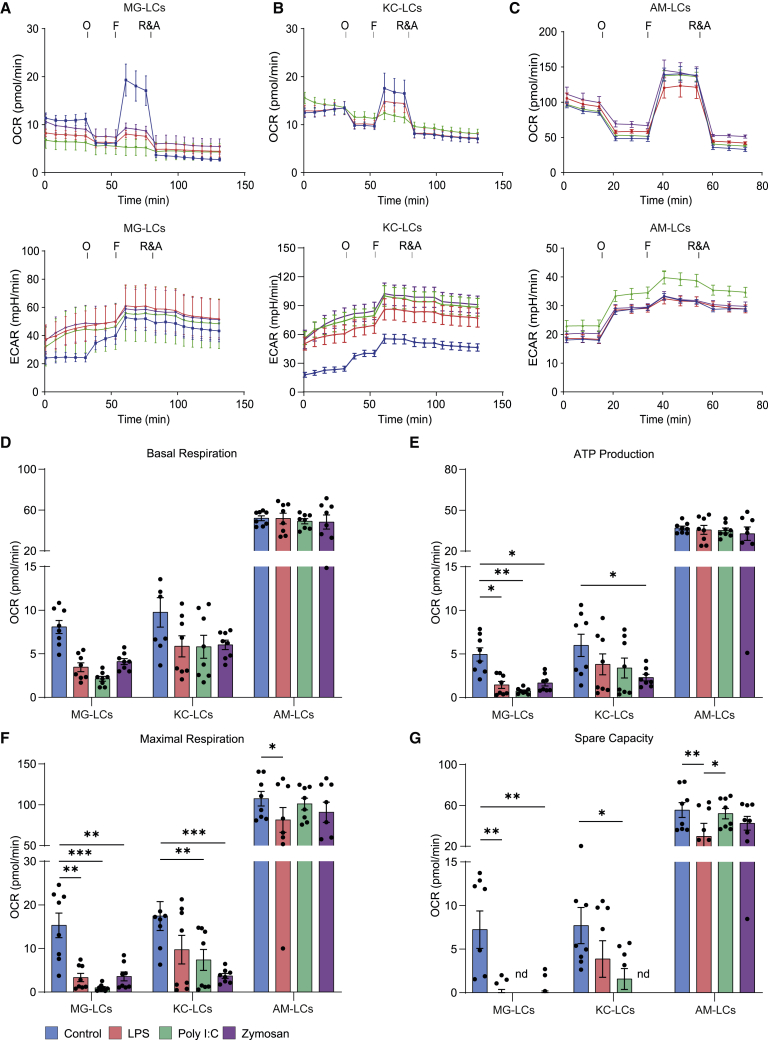
TRM-LCs employ different immunometabolic profiles upon stimulation *in vitro* (A–C) Bioenergetic profiles of TRM-LCs are presented. Oxygen consumption rate (OCR) (top) and extracellular acidification rate (ECAR) (bottom) are measured over time during oligomycin (O), carbonyl cyanide-p-trifluoromethoxyphenylhydrazone (F), and antimycin A plus rotenone (R&A) injections of MG-LCs (A), KC-LCs (B), and AM-LCs (C). Mean ± SEM of two independent experiments is shown; n = 8/group. (D–G) Quantification of basal respiration (D), ATP production (E), maximal respiration (F), and spare capacity (G) is presented. Mean ± SEM is shown; n = 8/group. Color code is indicated in the key. ∗p < 0.05, ∗∗p < 0.01, and ∗∗∗p < 0.001.

Finally, we tested if TRM-LCs can be useful to study immunometabolism and characterized TRM-LCs in mitochondrial stress tests. We compared untreated MG-, KC-, and AM-LCs with LPS-, poly(I:C)-, and zymosan-treated cells (Figure 7). In general MG- and KC-LCs showed a change in their oxygen consumption rate (OCR) upon immune stimulation compared with untreated cells (Figures 7A and 7B), whereas AM-LCs showed no alteration in their OCR (Figure 7C). Basal respiration remained unchanged in all TRM-LCs after immune stimulation (Figure 7D). ATP production, maximal respiration, and spare respiratory capacity were all reduced after immune stimulation. Maximal respiration dropped after immune stimulation in both MG- and KC-LCs, while this effect was much more pronounced in the former (Figures 7E–7G). In line with the above-described changes, we found an increase in the extracellular acidification rate (ECAR) in KC-LCs, but barely any changes in MG- and AM-LCs. The data reflect a decrease in oxidative phosphorylation in MG- and KC-LCs upon immune stimulation, whereas AM-LCs relied on oxidative phosphorylation even after immune stimulation. These data are in line with previous studies on their *ex vivo* counterparts. Furthermore, we found a clear switch of KC-LCs to glycolysis indicated by an increase in ECAR. Overall, the TRM-LCs represent a useful improved surrogate system to study the metabolism specific to each TRM population and might offer a useful screening tool for future studies.

## Discussion

Cell culture approaches for adult TRMs are an essential scientific tool to decipher their functions across different tissues. As of yet, understanding adult TRM functions in mechanistic detail has been hindered by the lack of adequate culture models that are able to capture major features of the macrophages *in vivo* while offering a sufficient cell yield. The system presented here addresses several of these technical obstacles, such as the easy cultivation of adult TRM-LCs without prior cell sorting. Our system also paves the way for follow-up functional studies elucidating the similarities and differences between these highly specialized cells.

Other cell culture approaches demonstrate a higher cell yield, avoiding FACS or MACS purification, but mostly rely on a combinatory culture of host tissue cells and TRMs and do not allow long-term culture of TRMs (Kitani et al., 2010; Li et al., 2014; Sepulveda-Diaz et al., 2016). Many mixed-culture protocols use early postnatal or embryonic tissues and are consequently suboptimal for the study of age-related defects in TRMs (Hassan et al., 1991; Honda et al., 1992; Shimizu et al., 2019). As prior enrichment for TRMs by cell sorting is not necessary in our system, it is an especially attractive model to avoid potential pre-activation. Instead, our system uses a specialized coating, namely PEI, which has previously been shown to favor the survival of neonatal CNS-resident MG in favor of other glia such as astrocytes (Sepulveda-Diaz et al., 2016). Here, we demonstrated that this coating strategy can be applied for various other adult TRMs as well.

Host-tissue-derived factors are essential to maintain TRMs efficiently in culture, and previous attempts to culture TRMs in the absence of these factors have consistently resulted in pre-activation and loss of tissue identity (Bohlen et al., 2017). Even though the cultures were devoid of any host cells after 2 weeks, our protocol enriched the culture medium with host-cell-derived factors during plating and due to the partial medium change. Thus, TRM-LCs were able to grow in the presence of various host-tissue-derived factors. Supplementation of the cultures with growth factors such as M-CSF, IL-34, or GM-CSF—depending on their host tissue—further promoted the partial preservation of the unique TRM phenotypes *in vitro*, confirming the importance of host tissue-derived growth factors for the survival of cultured macrophages (Busch et al., 2019; Butovsky et al., 2014). In addition, the oxygen levels used to obtain TRM-LCs are adjusted to organotypic levels. Atmospheric oxygen levels can inadvertently cause macrophage activation in culture, as hyperoxia is known to affect the activation stage of macrophages (Grodzki et al., 2013; Stuart et al., 2018; Tiede et al., 2011). Therefore, we suggest that the tissue normoxic conditions support the long-term cultivation of TRM-LCs and maintenance of their tissue-specific gene signature.

Even though we demonstrated preserved signature genes and surface-marker expression across the TRM-LCs, we detected certain alterations compared with their *ex vivo* isolated counterparts. Transcriptomic profiling showed the preservation of a core macrophage signature, but several genes, including signature genes of the specific TRM, are regulated *in vitro*. Hence, we cannot copy and preserve a full TRM phenotype with our organotypic protocol, but nevertheless provide a superior culture system to study functional intricacies across different TRM subsets compared with using BMDMs as an all-purpose solution. We found that TIM-4 is downregulated in KC-LCs, which could be due to the lack of endothelial cells and hepatocytes in the culture. Similarly, it was shown that MG lose the expression of surface markers such as the purinergic receptor P2ry12 or transmembrane protein 119 (Tmem119) in the absence of astrocytes and neurons (Bohlen et al., 2017; Gosselin et al., 2014; Van Hove et al., 2019). Cell culture medium and its supplementation can also affect the marker expression and differentiation of cultured macrophages (Kawakami et al., 2016), as previously reported for cultured BMDMs (Feuerstein et al., 2019), but also for AMs and MG *in vitro*. We further verified that the culture conditions used do not induce a TRM-specific macrophage signature in BMDMs, but rather maintain the host-tissue signature. More generally, our cultured TRM-LCs develop a higher granularity and autofluorescence *in vitro*, which correlates with the observed clearance of debris in the culture flasks as seen in live imaging (Videos S1 and S2). Furthermore, increases in inclusions and autofluorescence have been reported before in other cell culture approaches for TRMs such as MG of the CNS (Sepulveda-Diaz et al., 2016). Our organotypic culture system addresses many limitations of *in vitro* investigations and offers an improved surrogate system to study TRM function.

Changes in the signature of TRM-LCs seen *in vitro* could also be due to the fact that the cells are derived from another hematopoietic source compared with TRMs *in vivo*. As shown before for MG and KCs *in vivo*, their gene signature changes as soon as these cells are replaced by monocyte-derived cells (Scott et al., 2016; Shemer et al., 2018). We tested a potential contribution of monocyte-derived macrophages in our TRM-like cultures but could not find any evidence for this. However, we cannot exclude a potential contribution from tissue intrinsic progenitors rather than *bona fide* TRM populations in the respective organs to the pool of cells *in vitro*.

Functional differences in phagocytic capacity as well as cytokine and chemokine expression upon immune stimulation could be explored between the cultured TRM-LCs. However, as previously shown for MG and other TRMs such as PMs, altered culture conditions (e.g., supplementing the medium with FCS) can affect the phagocytosis capacity of the cells and the secretion of cytokines (Grace et al., 1988). To exclude cell-culture-induced artifacts, further comparison between our described *in vitro* phagocytosis rates and the phagocytosis rates of the different TRMs *ex vivo* would be needed. Nonetheless, it is evident that this system can be utilized in functional analyses in various assays, and therefore can serve as a useful tool to perform signaling studies, screening studies for pharmaceutical inhibitors or small molecules, etc. Accordingly, our organotypic cell culture system presents the possibility of gaining mechanistic insights *in vitro* that complement *in vivo* studies of TRMs.

In contrast to other cell culture protocols that rely on iPSCs or immortalized cell lines, our organotypic cell culture system offers the possibility of culturing TRM-LCs from transgenic mouse lines. In doing so, it provides the subsequent possibility of exploring the effects of gene deletions and genetic modifications *in vitro*. These applications could be of particular interest to the emerging field of immunometabolism, where higher cell numbers are often required for functional metabolic analyses. Given the range of possible applications for TRMs cultured in the organotypic system presented here, there is evidently a wide breadth of knowledge that can be gained from its utilization.

### Limitations of the study

As discussed above, we cannot yet overcome the limitations that TRM-LCs still show alterations in culture compared with their *ex vivo* counterparts. Furthermore, we cannot exclude that TRM-LCs are not actually derived from *bona fide* TRMs but rather from unknown tissue endogenous progenitors. Although our organotypic cell culture method results in high cell numbers of TRM-LCs, they seem to represent only the dominant resident TRM populations across different tissues; our model does not allow the specific enrichment or maintenance of smaller TRM populations found in host tissues, such as capsular macrophages in the liver or CNS-associated macrophages. For example, the culture system preferentially supported the growth of LPM-LCs over SPM-LCs. This could indicate that our system is most suitable for culturing long-lived TRMs that are maintained by endogenous proliferation and not TRMs that are short-lived and exchanged by HSC-derived progenitors.

## STAR★Methods

### Key resources table

**Table:** 

REAGENT or RESOURCE	SOURCE	IDENTIFIER
**Antibodies**		

Brilliant Violet 605 anti-mouse/human CD11b Antibody, Clone M1/70	BioLegend	Cat#101257; RRID: AB_2565431
Brilliant Violet 711™ anti-mouse CD11 c Antibody, Clone N418	BioLegend	Cat#117349; RRID: AB_2563905
Brilliant Violet 421™ anti-mouse CD115 (CSF-1R) Antibody, Clone AFS98	BioLegend	Cat# 135,513; RRID: AB_2562667
BUV395 Rat Anti-Mouse Siglec-F, Clone E50-2440	BD Biosciences	Cat# 740280; RRID: AB_2740019
BUV395 Rat Anti-Mouse Gr-1, Clone RB6-8C5	BD Biosciences	Cat# 563849; RRID: AB_2738450
CD45 Monoclonal Antibody (30-F11), APC-eFluor 780, eBioscience	Thermo Fisher Scientific	Cat# MCD4505; RRID: AB_10376146
PE/Cyanine7 anti-mouse F4/80 Antibody	BioLegend	Cat# 123114; RRID: AB_893478
PE anti-mouse CD170 (Siglec-F) Antibody, Clone S17007L	BioLegend	Cat# 155505; RRID: AB_2750234
Alexa Fluor 488 anti-mouse I-A/I-E Antibody, Clone M5/114.15.2	BioLegend	Cat# 107616; RRID: AB_493523
Alexa Fluor 700 anti-mouse I-A/I-E Antibody, Clone M5/114.15.2	Thermo Fisher	Cat # 56-5321-82; RRID: AB_494009
PE anti-mouse Tim-4 Antibody, Clone RMT4-54	BioLegend	Cat# 130006; RRID: AB_2201843
APC anti-mouse Tim-4 Antibody, Clone RMT4-54	BioLegend	Cat# 130021; RRID: AB_2892285
Brilliant Violet 421™ anti-mouse CD115 Antibody, Clone AFS98	BioLegend	Cat#135513; RRID:AB_2562667
Brilliant Violet 421™ anti-mouse CD45, Clone 30-F11	BioLegend	Cat# 103133; RRID: AB_10899570
PE anti-mouse CD115, Clone AFS98	BioLegend	Cat# 135505; RRID: AB_1937254
PE anti-mouse Ly-6G/Ly-6C (Gr-1), Clone RB6-8C5	BioLegend	Cat# 108407; RRID: AB_313372
Biotin anti-mouse Ly-6G/Ly-6C (Gr-1) Antibody	BioLegend	Cat# 108403; RRID: AB_313368
eBioscience Fixable Viability Dye eFluor 780	Thermo Fisher Scientific	Cat# 65-0865-14
Anti-mouse/human Iba1, Rabbit	FUJIFILM Wako Pure Chemical Corporation	Cat# 019-19741; RRID: AB_839504
Goat anti-Rabbit IgG (H+L) Highly Cross-Adsorbed Secondary Antibody, Alexa Fluor 568	Thermo Fisher Scientific	Cat# A-11036; RRID: AB_10563566
Griffonia Simplicifolia Lectin I (GSL I) isolectin B4, Fluorescein	Vector Laboratories	Cat# FL1201; RRID: AB_2314663
rat Anti-CD68 (mouse) antibody, Clone FA-11	Abcam	Cat# ab53444
Donkey Anti-Rat IgG H&L (Alexa Fluor® 568) preadsorbed	Abcam	Cat# ab175475
Anti-Biotin MicroBeads	Miltenyi Biotec	Cat# 130-090-485
Precision Count Beads	BioLegend	Cat# 424902

**Chemicals, peptides, and recombinant proteins**		

DAPI (4',6-Diamidino-2-Phenylindole, Dilactate)	Thermo Fisher Scientific	Cat# D3571; RRID: AB_2307445
pHrodo Red Zymosan Bioparticles	Thermo Fisher Scientific	P35364
Cytochalasin D	Sigma-Aldrich	C2618
Opti-MEM	Thermo Fisher Scientific	11058021
Trypsin-EDTA (0,05%), phenol red	Thermo Fisher Scientific	25300062
Polyethylenimine (PEI)	Sigma-Aldrich	408727
Fetal calf serum (FCS)	Thermo Fisher Scientific	Cat#10270106
Recombinant Murine CSF-1	Peprotech	Cat# 315-02
Recombinant Murine IL-34	BioLegend	Cat# 577606
Recombinant Murine GM-CSF	Peprotech	Cat# 315-03
Percoll	Sigma-Aldrich	Cat# P4937
Poly I:C	Tocris	Cat# 7414
Lipopolysaccharides	Sigma-Aldrich	297-473-0
Zymosan	Sigma-Aldrich	58856-93-2
Borax solution	Sigma-Aldrich	24895881

**Critical commercial assays**		

Biozym Blue S’Green qPCR Kit	Biozym	331416XL
LEGENDplex™ Multiplex Assays	BioLegend	740845
High Capacity RNA-to-cDNA Kit	Applied Biosciences	10704217
Seahorse XF Cell Mito Stress Test Kit	Agilent	103015-100
Rneasy Mini Kit	QIAGEN	74004
QuadroMACS™ Separator and Starting Kits	Miltenyi	130-091-051

**Deposited data**		

Bulk RNA-sequencing data set	This paper	ID GSE196376

**Experimental models: Organisms/strains**		

Mouse: *C57BL/6J*	Charles River Laboratories	RRID: IMSR_JAX:000664

**Oligonucleotides**		

PRIMER: *Id3* forward: 5‘-ttgtgatctccaaggacaagagg-‘3	This paper	N/A
PRIMER: *Id3* reverse: 5‘-gtaagtgaagagggctgggttaa-‘3	This paper	N/A
PRIMER: *Nr1h3* forward: 5‘-tcaatgcctgatgtttctcctga-‘3	This paper	N/A
PRIMER: *Nr1h3* reverse: 5‘-gactccaaccctatccctaaagc-‘3	This paper	N/A
PRIMER: *Car4* forward: 5‘-caaatgggaatgacaacggttca-‘3	This paper	N/A
PRIMER: *Car4* reverse: 5‘-tagaggttgaatggggtttggag-‘3	This paper	N/A
PRIMER: *Siglecf* forward: 5‘-aaccctgcctccaccataaatag-‘3	This paper	N/A
PRIMER: *Siglecf* reverse: 5‘-acagatttcatagcctccgtgtt-‘3	This paper	N/A
PRIMER: *Cebpb* forward: 5‘-ccttataaacctcccgctcgg-‘3	This paper	N/A
PRIMER: *Cebpb* reverse: 5‘-gctcgtagtagaagttggccac-‘3	This paper	N/A
PRIMER: *Tgfb2* forward: 5‘-tgcaatgctgtgggagaagt-‘3	This paper	N/A
PRIMER: *Tgfb2* reverse: 5‘-ccagcactcggtcaaagtct-‘3	This paper	N/A
PRIMER: *Fcrls* forward: 5‘-cttgtgaggctgaaaacgcc-‘3	This paper	N/A
PRIMER: *Fcrls* reverse: 5‘-gccattcaccaaacgcactt-‘3	This paper	N/A
PRIMER: *Gapdh* forward: 5‘-gggttcctataaatacggactgc-‘3	This paper	N/A
PRIMER: *Gapdh* reverse: 5‘-ccattttgtctacgggacga-‘3	This paper	N/A
PRIMER: *Hexb* forward: 5‘-ctggtgtcgctgtcgc-‘3	This paper	N/A
PRIMER: *Hexb* reverse: 5‘-cagggccatgctcttg-‘3	This paper	N/A
PRIMER: *Ccl2* forward: 5’- catccacgtgttggctca-‘3	This paper	N/A
PRIMER: *Ccl2* reverse: 5’- gatcatcttgctggtgaatgagt-‘3	This paper	N/A
PRIMER: *Il1b* forward: 5’- ttgacggaccccaaaagat-‘3	This paper	N/A
PRIMER: *Il1b* reverse: 5’- gaagctggatgctctcatctg-‘3	This paper	N/A
PRIMER: *Il6* forward: 5’- gctaccaaactggatataatcagga-‘3	This paper	N/A
PRIMER: *Il6* reverse: 5’- ccaggtagctatggtactccagaa-‘3	This paper	N/A
PRIMER: *Tnfa* forward: 5’- cagaaatgagagaggcatgaga-‘3	This paper	N/A
PRIMER: *Tnfa* reverse: 5’- gcttgatctcccgttatctcc-‘3	This paper	N/A

**Software and algorithms**		

BD FACSDiva	BD Biosciences	https://www.bdbiosciences.com/en-us/instruments/research-instruments/research-software/flow-cytometry-acquisition/facsdiva-software
FlowJo	Tree Star	https://www.flowjo.com/solutions/flowjo
Wave	Agilent	https://www.agilent.com/en/product/cell-analysis/real-time-cell-metabolic-analysis/xf-software/seahorse-wave-desktop-software-740897
ImageJ	ImageJ	https://imagej.nih.gov/ij/
GraphPad Prism	GraphPad	https://www.graphpad.com/
Incucyte® Software (v2019B)	Essen BioScience	https://www.essenbioscience.com/de/products/software/incucyte-software-v2019b/
LAS X	Leica	https://www.leica-microsystems.com/de/produkte/mikroskop-software/p/leica-las-x-ls/

### Resource availability

#### Lead contact

Further information and requests for resources and reagents should be directed to and will be fulfilled by the lead contact: Katrin Kierdorf, katrin.kierdorf@uniklinik-freiburg.de.

#### Materials availability

This study did not generate new unique reagents.

### Experimental model and subject details

#### Mice

*C57BL6/J* mice (Charles River): 8 weeks old, female, co-housed, 12 h/12 h light-dark cycle, water and food *ad libitum*.

*Ccr2*^*−/−*^ mice (*B6.129S4-Ccr2*^*tm1Ifc*^*/J*) (Jackson Laboratory (#004999)): 8 weeks old, female and male, backcrossed to C57BL6/J mice, co-housed, 12 h/12 h light-dark cycle, water and food *ad libitum*.

All animal experiments and protocols were performed in accordance with the respective national, federal and institutional regulations and were approved by Regierungspräsidium Freiburg (X-17/02A, G-20/42). All possible efforts were made to minimize animal suffering and the number of animals used.

### Method details

#### Mice

Adult 8 weeks old female *C57BL6/J* mice (Charles River) were used as wildtype (WT) mice throughout all experiments. *Ccr2*^*−/−*^ mice (*B6.129S4-Ccr2tm1Ifc/J*) were originally obtained from the Jackson Laboratory (#004999) and backcrossed to C57BL6/J mice. Mice were co-housed and kept at 12 h/12 h light-dark cycle with water and food *ad libitum*.

#### Transcardial perfusion

Adult tissues for cell culture were isolated from 8 weeks old female C57BL6/J mice after transcardial perfusion with phosphate buffered saline PBS (Sigma Aldrich). Mice were anaesthetized with a 200 μL intraperitoneal injection of Ketamine (10%, Essex Tierarznei) and Xylazin ((Rompun) 2%, Bayer) (100 mg/kg Ketamine and 10 mg/kg Xylazin). After sterilizing the skin with 70% ethanol the mice were placed under a sterile laminar flow hood. Mice were sacrificed and transcardially perfused with 10 mL ice-cold PBS.

#### Brain dissection and preparation for cell culture

The whole brain was removed from the skull and transferred into a glass potter filled with 10 mL of cold dissection medium (Hank’s Balanced Salt Solution (HBSS, Thermo Fisher Scientific), 1,5% HEPES (1 M) (Thermo Fisher Scientific) and 1,3% Glucose (45%, Sigma-Aldrich)). The brain was homogenized in dissection medium to a single cell suspension and transferred through a 70 μm cell strainer (Corning) into a 50 mL tube (Greiner). Afterwards the tube was centrifuged for 10 min at 400 g and 4°C. The supernatant was discarded and the cell pellet was resuspended in 10 mL of 37% Percoll (Sigma-Aldrich) and transferred into a fresh 15 mL tube (Greiner). The density gradient was centrifuged for 30 min at 1000 g at 4°C without brake and the resulting cell pellet contained the cells of interest. Afterwards cells were kept on ice and the myelin was carefully removed with the supernatant leaving only 1 mL on top of the cell pellet to increase isolation efficiency. The cell pellet was resuspended using a P1000 pipette and transferred with the same tip into a fresh 15 mL tube to decrease myelin contamination. The cells were washed by adding 10 mL PBS and then again centrifuged at 300 g for 5 min at 4°C. Supernatant was discarded and the cell pellet was resuspended in 10 mL MG medium. The cell suspension was then plated into previously coated T25 flasks (TPP). Three brains were cultured per flask.

#### Liver dissection and preparation for cell culture

The liver was removed followed by 3 consecutive baths in sterile PBS. Next, the liver was transferred into 1 mL of enzyme mix (PBS, 4% fetal calf serum (FCS) (Thermo Fisher Scientific), 2 mg/mL DNase (Sigma Aldrich), 1 mg/mL Collagenase (Sigma Aldrich)) and cut into small pieces. The liver was digested in this enzyme mix for 30 min at 37°C. Afterwards, 1 mL of FACS buffer (PBS, 5% FCS, 2 mM EDTA) was added to stop the enzymatic reaction. The suspension was transferred into a 50 mL tube and passed through a 100 μm strainer (Corning) via mechanical dissociation while washing with 9 mL FACS buffer. The cells were then centrifuged at 400 g for 10 min at 4°C after which the supernatant was discarded. The cell pellet was resuspended in 10 mL of 37% Percoll and transferred into a fresh 15 mL tube. The gradient was centrifuged for 30 min at 1000 g at 4 °C without brake. Afterwards the cell pellet was kept on ice and the supernatant was carefully removed leaving 1 mL behind to increase the cell yield. The cell pellet was resuspended and transferred to a fresh 15 mL tube. PBS was added in order to dilute the remaining Percoll and to wash the cells. Cells were centrifuged at 300 g for 5 min at 4°C. The supernatant was discarded and the cell pellet was resuspended in 1 mL of red blood cell (RBC) lysis buffer (MilliQ water, 0.1 M NH_4_Cl, 19 mM NaHCO3, 0.1 mM EDTA). The cell suspension was incubated on ice for 5 min. Subsequently, 9 mL FACS buffer were added and the suspension was mixed by inversion. Cells were centrifuged at 300 g for 5 min at 4°C and the supernatant was discarded. The cell pellet was resuspended in 10 mL KC medium and plated in previously coated T25 flasks. One liver was cultured per flask.

#### Lung dissection and preparation for cell culture

The lung was removed followed by 3 consecutive baths in sterile PBS. The lung was transferred into 1 mL of enzyme mix (PBS, 4% FCS, 2 mg/mL DNase, 1 mg/mL Collagenase), cut into small pieces and digested in the enzyme mix for 30 min at 37°C. 1 mL FACS buffer was added to stop the enzymatic reaction and the lung was transferred into a 50 mL tube through a 100 μm strainer by mechanical dissociation while washing with 9 mL FACS buffer (PBS, 5% FCS, 2 mM EDTA). The cells were centrifuged at 400 g for 10 min at 4°C and the supernatant was discarded. Cell pellet was resuspended in 10 mL of 37% Percoll and transferred to a fresh 15 mL tube. The density gradient was centrifuged for 30 min at 1000 g at 4°C without brake, the cell pellet contained the cells of interest. Afterwards cells were kept on ice and the supernatant was carefully removed leaving 1 mL supernatant and the cell pellet in the tube for maximal cell yield. The cell pellet was then resuspended and transferred into a fresh 15 mL tube. 10 mL PBS were added in order to dilute the remaining Percoll and wash the cells. Cells were centrifuged at 300 g for 5 min at 4°C. The supernatant was discarded and the cell pellet resuspended in 1 mL of RBC lysis buffer. Suspension was put on ice for 5 min. Subsequently 9 mL FACS buffer were added and the suspension was mixed by inversion. The cells were centrifuged at 300 g for 5 min at 4°C and the supernatant was discarded. The cell pellet was resuspended in 10 mL AM medium and plated in previously coated T25 flasks. Three lungs were cultured per flask.

#### Peritoneal lavage for cell culture

After sterilizing the skin with 70% ethanol the mice were placed under a laminar flow hood. Mice were sacrificed and the ventral skin was opened without opening the underlying peritoneum. 5 mL of PBS were injected (27-G needle, Sterican, B.Braun) into the peritoneum. PBS was drawn out through the peritoneum with a fresh syringe (20-G needle, Sterican, B.Braun). Cells were centrifuged for 5 min with 400 g at 4°C. The supernatant was removed before resuspending the cell pellet in 10 mL PM medium. The cells were plated in previously coated T25 flasks. Cells from the peritoneal cavity of three mice were cultured per flask.

#### Isolation of bone marrow for cell culture

After sterilizing the skin with 70% ethanol the mice were placed under a laminar flow hood. The hind legs were dissected and femur and tibia were collected. To be sure that bones are not damaged, the hind legs were cut off at the trochanter major proximal of the acetabulum. Bones were kept in PBS on ice until further preparation. Femur and tibia were separated from each other, the fibula and tendons were removed. Prepared bones were then shorty washed in 70% ethanol and afterwards in PBS. Subsequently they were opened with bone scissors on both sides and the bone marrow was flushed out with 20 mL PBS using a syringe and passed through a 100 μm cell strainer into a 50 mL tube. Cells were centrifuged for 5 min with 400 g at 4°C. The supernatant was discarded and the cells were resuspended in BMDM media. Cells from one leg were cultured per Petri dish.

#### Tissue macrophage cell culture

TRM-LCs were cultured in T25 Flasks. T25 flasks were coated with 1 mg/mL polyethylenimine (PEI) (in a pH 8.3 borate buffer) 1 h at 37°C in order to obtain better attachment of the macrophages to the plate, afterwards the flasks were washed three times with PBS and cells were plated. Cells were cultured with 10 mL of the corresponding medium. Adult MG-LCs and BMDMs (hypoxia M-CSF & IL-34) were cultured in DMEM containing 4.5 g/L D-Glucose and L-Glutamine (Thermo Fisher Scientific) and supplemented with 10% heat inactivated FCS, 1% Penicillin-Streptomycin (P/S, Sigma-Aldrich), 20 ng/mL recombinant murine M-CSF (Peprotech) and 20 ng/mL recombinant murine IL-34 (BioLegend). PM-LCs, KC-LCs and BMDMs (hypoxia & normoxia M-CSF) were cultured in DMEM containing 4.5 g/L D-Glucose and 4.5 g/L L-Glutamine and supplemented with 10% FCS, 1% P/S, 20 ng/mL recombinant murine M-CSF. AM-LCs were cultured in DMEM containing 4.5 g/L L-Glutamine (Thermo Fisher Scientific) and supplemented with 10% FCS, 1% P/S, 20 ng/mL recombinant murine GM-CSF (Peprotech). BMDMs (normoxia GM-CSF) were cultured in DMEM containing 4.5 g/L D-Glucose and L-Glutamine and supplemented with 10% FCS, 1% P/S, 20 ng/mL recombinant murine GM-CSF. MG-, KC-, PM-LCs and BMDMs (hypoxia) were cultured in a hypoxic chamber (Stemcell technologies) flooded with a gas mixture composed of 3% O_2_, 5% CO_2_ and 92% N_2_ in an incubator at 37°C. AM-LCs and BMDMs (normoxia) were placed in an incubator with atmospheric oxygen levels and 5% CO_2_ at 37°C. 2/5 of the medium was changed on day two of culture inside a laminar flow hood for alveolar macrophages and inside a hypoxia workstation (Whitley H35 Hypoxystation, Mmeintrup dwsDWS) for MG-, KC- and PM-LCs. The cells were cultured without further media change for 12 further days.

#### MACS depletion of Gr1^+^ cells before culture

To deplete Gr1^+^ cells before culture, magnetic activated cell sorting (MACS) was performed. MACS sorting was performed according to manufacture manual. In brief, isolated cells were resuspended in 500 μL PBS and pre-treated with Fc block (1:200) for 15 min (final concentration: 40 ng/μL). All cells were treated with 4 μL of biotinylated anti-mouse Ly6G/Ly6C (Gr1) for 15 min. Afterwards cells were washed with 2 mL FACS buffer and centrifuged at 300 g for 10 min. The supernatant was carefully discarded. After repeating the washing step, cells were resuspended in 70 μL FACS buffer and magnetically labeled with 30 μL of anti-biotin Micro-Beads. For the cell pellets of the liver, 50 μL of anti-biotin Micro-Beads were added. The cells were incubated for 15 min at 4 °C. Then, the cells were washed with 2 mL FACS buffer and centrifuged at 300 g for 10 min. The supernatant was discarded, and the cells were resuspended in 500 μL of FACS buffer before the magnetic separation using LS Columns was performed. Prior applying the cell suspension, LS Columns were placed in the MACS QuadroSeparator and rinsed with 3 mL FACS buffer. To deplete Gr1^+^ cells, the cell suspension was applied onto the LS Columns and the flow-through was collected. Afterwards the columns were washed three times with 3 mL FACS buffer and again the flow-through was collected. Subsequently the flow-through was centrifuged at 300 g for 10 min and the supernatant was discarded. The cell pellet was resuspended in corresponding media and the cells were cultured as described above. To analyze the depletion efficiency 1:10 of the cells as well as cells which were not MACS sorted were stained as described in *“*Flow cytometry of TRM*”*.

#### Cytospins and Pappenheim staining

To perform cytospins, cells were detached from the culture flasks with a trypsin-EDTA solution (0,05% porcine trypsin; 0,02% EDTA (Sigma-Aldrich) and incubated for 5 min at 37°C. 9 mL FACS buffer was added to block the enzymatic reaction and the detached cell suspension was transferred into a fresh 15 mL tube (Greiner) and centrifuged at 300 g for 5 min 500 μL of the obtained cell suspension containing 100.000 cells were added to a cytofunnel of a cytocentrifuge. Cells were centrifuged at 600 rpm for 5 min to spin the cells down on a slide. The supernatant was discarded and the slides were centrifuged at 1100 rpm for 3 min. Slides were air dried prior to staining. To perform Pappenheim staining, slides were stained for 4 min with May-Grünwald stain (Merck), MilliQ water was added and the slides were incubated for 4 min. Slides were washed with MilliQ water and Giemsa staining (Merck) (1:10) was performed for 10 min. Afterwards the slides were washed with MilliQ water and a coverslip was mounted.

#### Immunocytochemistry

Cultured cells were detached with trypsin-EDTA solution as described above. 100.000 cells were plated in an 8-well ibidi® chamber slide (ibidi) in 300 μL of corresponding culture media. The medium was removed after one day and the cells were washed with cold PBS. Freshly thawed 4% paraformaldehyde (PFA) was added for 1 h at 4 °C to fix the cells. PFA was removed and the fixed cells were washed three times with PBS. Cells were blocked with blocking buffer (10% bovine serum albumin (BSA) (Sigma-Aldrich), 0.1% Triton-X) for 1 h at room temperature. The blocking solution was removed, and the cells were either stained with an anti-Iba1 antibody (polyclonal rabbit anti-mouse, 2.5 μg/mL, Wako) diluted in blocking buffer over night at 4°, or cells were stained with an anti- CD68 antibody (monoclonal rat anti-mouse, 2 μg/mL, Abcam) and isolectin-B4 (Griffonia Simplicifolia Lectin I (GSL I) isolectin-B4, Fluorescein, 2 μg/mL, Vector Laboratories) over night at 4°C. The culture slides were then washed three times with PBS. The secondary antibodies AlexaFluor™568-conjugated donkey anti-rabbit (3 μg/mL, Thermo Fisher Scientific) or AlexaFluor™568-conjugated donkey anti-rat (2 μg/mL, Abcam) and DAPI (1:5000, Sigma-Aldrich), added as a nuclear staining, were diluted in blocking solution and incubated on the slides for 2 h at room temperature. Afterwards cells were washed three times with PBS and kept in the dark at 4°C until imaging. Samples were imaged using a confocal microscope (SP8X with WLL, Leica).

#### Flow cytometry of TRM

For flow cytometric analysis of freshly *ex vivo* isolated TRM, we performed flow cytometry of the obtained cell pellets after Percoll gradient. The supernatant was discarded until only the cell pellet and 50 μL supernatant remained. Cells were stained as described below. For flow cytometric analysis of *in vitro* cultured TRM-LCs, cells were detached from plates with trypsin-EDTA solution as described above. 9 mL FACS buffer was added to block the enzymatic reaction and the detached cell suspension was transferred into FACS tubes and centrifuged at 300 g for 5 min. The supernatant was discarded until only the cell pellet and 50 μL supernatant remained. The (Fc) receptor of the cells was blocked by adding a monoclonal mouse anti-CD16/CD32 antibody (Biolegend) diluted 1:200 in PBS for 20 min. Cells were stained afterwards for 20 min on ice in the dark by adding direct-labeled antibodies in a 1:200 dilution in PBS. All macrophages were stained with the following antibodies: APC-labeled anti-CD45 (30-F11, eBioscience), BV605-labeled anti-CD11b (M1/70, BioLegend), FITC-labeled anti-MHC-II (M5/114.15.2, BioLegend), PE-labeled anti-TIM-4 (RMT4-54, BioLegend), BUV395-labeled anti-SiglecF (E50-2440, BD), BV421-labeled anti-CD115 (AFS98, BioLegend), PE-Cy7-labeled anti-F4/80 (BM8, BioLegend) and BV711-labeled anti-CD11 c (N418, BioLegend). To determine the cell composition in the pellet after the Percoll gradient the cells were stained with individual panels. MG pellets were stained with the following antibodies: APC-labeled anti-CD45 (30-F11, eBioscience), BV605-labeled anti-CD11b (M1/70, BioLegend), AF700-labeled anti-MHC-II (M5/114.15.2, Thermo Fisher), PE-labeled anti-Gr1 (RB6-8C5, BioLegend), BV421-labeled anti-CD115 (AFS98, BioLegend). KC pellets were stained with the following antibodies: BV605-labeled anti-CD11b (M1/70, BioLegend), PE-Cy7-labeled anti-F4/80 (BM8, BioLegend), AF700-labeled anti-MHC-II (M5/114.15.2, Thermo Fisher) BV41-labeled anti-CD45 (30-F11, BioLegend), BUV395-labeled anti-Gr1 (RB6-8C5, BD Biosciences), PE-labeled anti-CD115 (AFS98, BioLegend), APC-labeled anti-TIM-4 (RMT4-54, BioLegend). PM pellets were stained with the following antibodies: APC-labeled anti-CD45 (30-F11, eBioscience), BV605-labeled anti-CD11b (M1/70, BioLegend), AF700-labeled anti-MHC-II (M5/114.15.2, Thermo Fisher), PE-labeled anti-Gr1 (RB6-8C5, BioLegend), BV421-labeled anti-CD115 (AFS98, BioLegend), PE-Cy7-labeled anti-F4/80 (BM8, BioLegend). AM pellets were stained with the following antibodies: APC-labeled anti-CD45 (30-F11, eBioscience), BV605-labeled anti-CD11b (M1/70, BioLegend), AF700-labeled anti MHC-II (M5/114.15.2, Thermo Fisher), PE-labeled anti-Gr1 (RB6-8C5, BioLegend), BV421-labeled anti-CD115 (AFS98, BioLegend), BUV-labeled anti-SiglecF (E50-2440, BD Biosciences). The cells were then washed with PBS and centrifuged. After centrifugation, the cell pellet was resuspended in 200 μL FACS buffer. In order to obtain relative cell numbers, 30μL Precision Count Beads™ were added to the corresponding sample right before measuring. To compensate the cytometer, compensation beads (Thermo Fisher) were stained in 400 μL FACS buffer using the same direct coupled antibodies individually at a dilution of 1:400 except for APC-Cy 7 where we used APC-Cy7-labeled anti-CD45 (30-F11, BioLegend). Fixable Viability Dye 780 (eBioscience) (1:1000) was added as a live/dead stain to the antibody mix. Cells were measured with a BD LSR Fortessa SORP flow cytometer and data was analyzed with FlowJo VX/V10.7.

#### FACS sorting of TRM

TRM from brain, liver, peritoneum and lung were FACS sorted for RNA-sequencing. Therefore TRM were isolated as described above and cells were stained for flow cytometry. The (Fc) receptors of the cells were blocked by adding a monoclonal mouse anti-CD16/CD32 antibody (Biolegend) diluted 1:200 in PBS for 20 min. Cells were stained afterwards for 20 min on ice in the dark by adding direct-labeled antibodies in a 1:200 dilution in PBS. MG were stained with the following antibodies: APC-labeled anti-CD45 (30-F11, eBioscience), BV605-labeled anti-CD11b (M1/70, BioLegend). KC were stained with the following antibodies: APC-labeled anti-CD45 (30-F11, eBioscience), BV605-labeled anti-CD11b (M1/70, BioLegend), PE-Cy7-labeled anti-F4/80 (BM8, BioLegend), PE-labeled anti-TIM-4 (RMT4-54, BioLegend). PM were stained with the following antibodies: APC-labeled anti-CD45 (30-F11, eBioscience), BV605-labeled anti-CD11b (M1/70, BioLegend), PE-Cy7-labeled anti-F4/80 (BM8, BioLegend), BV421-labeled anti-CD115 (AFS98, BioLegend). AM were stained with the following antibodies: APC-labeled anti-CD45 (30-F11, eBioscience), BV605-labeled anti-CD11b (M1/70, BioLegend), PE-labeled anti-SiglecF (S17007L, BioLegend). Fixable Viability Dye 780 (eBioscience) (1:1000) was added as a live/dead stain to the antibody mix. The cells were then washed with PBS and centrifuged. After centrifugation, the cell pellet was resuspended in 500 μL FACS buffer. TRM were sorted with a MoFlo Astrios with a 70 μm nozzle. Up to 50.000 TRM/sample were sorted into RLT buffer containing 1% β-mercaptoethanol and stored at −20°C until further analysis.

#### RNA-sequencing and RNA-sequencing analysis

RNA extraction, library preparation and RNA-sequencing were performed at the Genomics Core Facility “KFB - Center of Excellence for Fluorescent Bioanalytics” (University of Regensburg, Regensburg, Germany; www.kfb-regensburg.de. Total RNA was extracted from FACS sorted and cultured TRM-LCs and stabilized in RLT buffer according to the “Purification of total RNA from animal and human cells” protocol of the RNeasy Micro Kit (QIAGEN). In brief, cells were stored in buffer RLT containing 1% beta-mercaptoethanol and shipped on dry ice. After thawing the samples were homogenized by vortexing for 1 min. Next one volume of 70% ethanol was added and the samples were applied to RNeasy MinElute spin columns, followed by an on-column DNase digestion and several wash steps. Finally total RNA was eluted in 14 μL of nuclease free water. Purity and integrity of the RNA was assessed on the Agilent 2100 Bioanalyzer with the RNA 6000 Pico LabChip reagent set (Agilent). The SMARTer Ultra Low Input RNA Kit for Sequencing v4 (Clontech Laboratories) was used to generate first strand cDNA from approximately 1 ng total-RNA. Double stranded cDNA was amplified by LD PCR (11 cycles) and purified via magnetic bead clean-up. Library preparation was carried out as described in the Illumina Nextera XT Sample Preparation Guide (Illumina). Thereby 150 pg of input cDNA were tagmented (tagged and fragmented) by the Nextera XT transposome. The products were purified and amplified via a limited-cycle PCR program to generate multiplexed sequencing libraries. For the PCR step 1:5 dilutions of the unique dual indexing (i7 and I5) adapters were used. The libraries were quantified using the KAPA Library Quantification Kit - Illumina/ABI Prism User Guide (Roche). Equimolar amounts of each library were sequenced on an Illumina NextSeq 2000 instrument controlled by the NextSeq 2000 Control Software (NCS) v1.4.0.39521, using one 50 cycles P3 Flow Cell with the dual index, single-read (SR) run parameters. Image analysis and base calling were done by the Real Time Analysis Software (RTA) v3.9.2. The resulting.cbcl files were converted into.fastq files with the bcl2fastq v2.20 software. Single-end reads were trimmed, using Trimmomatic (v0.38)(Bolger et al., 2014), to remove adapter content and bad quality reads. Trimmed reads were aligned to the mouse reference genome (mm10) and reads-per-gene was quantified with STAR (v2.7.0a) (Dobin et al., 2013). Differential analysis was performed with the linear model-based approach limma R package (limma-voom)(Ritchie et al., 2015). The generally applicable gene set enrichment (GAGE) R package was used to identified enriched gene-sets from MSigDB(Luo et al., 2009; Subramanian et al., 2005). For both analyses, an adjusted p value (Benjamini-Hochberg) below 0.05 was considered as significant. RNA-seq data have been uploaded to Gene Expression Omnibus and are available under the ID GSE196376 using the token qnmraksexbgrrux.

#### Immune stimulation with LPS, poly(I:C), or zymosan *in vitro*

Cultured TRM-LCs were stimulated *in vitro* with either LPS (100 ng/mL), poly(I:C) (25 μg/mL) or zymosan (10 μg/mL) for 24 h.

#### Gene expression analysis

Cultured TRM-LCs were detached from plates with trypsin-EDTA as described above. 100.000 cells were centrifuged for 5 min at 300 g and supernatant was discarded. The pellet was resuspended in 350 μL RLT Buffer (Qiagen) containing 1% beta-mercaptoethanol. The suspension was transferred to a QIAshredder column (Qiagen) and centrifuged 2 min at full speed, afterwards the RNeasy Plus Micro Kit (Qiagen) was used according to the manufacturer’s protocol. To obtain cDNA the High-Capacity RNA-to-cDNA Kit (Applied Biosciences) was used according to the manufacturer’s protocol. The cDNA was stored in the freezer at −20°C until further use. To determine relative gene expression, semi-quantitative qPCR analysis was performed. All steps were performed on ice. The cDNA was diluted 1:10 to the working concentration. To make a dilution series, all cDNA samples were pooled as a standard sample followed by five consecutive 1:5 dilutions to obtain a standard curve. A standard curve to test primer efficiency was monitored for each qPCR run and each primer pair used. Gapdh was chosen as a housekeeping gene. For each well of a 96 qPCR plate (Biozym), 4 μL of cDNA of the target cells or of the standard sample and 16 μL of a qPCR mix containing 10 μL Sensimix (Biozym), 5,6 μL H2O and 0,4 μL Primermix (10 μM) were added to the corresponding wells. The qPCR plate was covered with adhesive clear qPCR seal (Biozym) followed by a short spin. The assay was performed using a LightCycler480 (Roche) and analyzed via the LightCycler Software (Roche). Target gene expression was normalized to the expression of the house keeping gene Gapdh. Not detectable gene expression was quantified as 0.

#### LEGENDplex cytokine assays

In order to measure cytokine secretion by untreated and treated TRM-LCs, 100.000 cells were replated in a 48-well plate in the corresponding medium as previously described. The cells were cultivated for additional 24 h, then stimulated with LPS (100 ng/mL), zymosan (10 μg/mL), or poly I:C (25 μg/mL) or PBS as control for 24 h. After 24 h the supernatant was removed and stored at −20°C until further analysis. For quantification of multiple cytokines the supernatants were analyzed using a bead-based immunoassay LEGENDplex-kit from BioLegend. The kit was used according to the “LEGENDplex™ Multi-Analyte Flow Assay Kit” protocol. Cytokines were measured with a BD LSR Fortessa SORP flow cytometer and data was analyzed with FlowJo VX/V10.7.

#### Live imaging

Organs were processed following the respective organ-specific protocol described above and plated into 6-well plates and cultured according organ-specific protocol. After 2 days of culture 2/5 of the media was changed and the plate was transferred into the Incucyte S3 Live-Cell Analysis System housed inside a cell incubator set at 37 °C, 5% CO_2_ until the assay was complete after 14 days. Four phase contrast images were taken from each well every 2 h using a 10× objective lens and then analyzed using the Incucyte™ Software (v2019B).

#### pHrodo Red Zymosan Bioparticles phagocytosis assay

In order to compare phagocytosis dynamics between the different TRM-LCs, phagocytosis of pHrodo Red Zymosan Bioparticles (Thermo Fisher) was analyzed. Cultured TRM-LCs were detached from plates as described above. The cells were counted using a Neubauer counting chamber and 100.000 cells were plated into Nunc 48 well plates (Thermo Fisher). After 20 min at room temperature the plate was transferred to a 37°C hypoxic incubator. After 24 h medium was replaced by Opti-MEM (Fisher Scientific) to starve the cells for 2 h. As a negative control, Cytochalasin D (100 μM, Sigma Aldrich) was added to negative control wells to inhibit phagocytosis and incubated for 45 min pHrodo Bioparticles were resuspended in Opti-MEM to 0.5 mg/mL and sonicated on ice three times for 20 s. Medium was removed from the wells and the suspension with the pHrodo Bioparticles was added. Four different time points were measured. The pHrodo Bioparticle suspension was removed after 5, 10, 15 or 30 min depending on the well. The cells were washed with PBS two times before detaching them. Cells were analyzed via flow cytometry as described above. The (Fc) receptor of the cells was blocked by adding a monoclonal mouse anti-CD16/CD32 antibody (Biolegend) diluted 1:200 in PBS for 20 min. Cells were stained afterwards for 20 min on ice in the dark by adding direct-labeled antibodies in a 1:200 dilution in PBS. All macrophages were stained with the following antibodies: APC-labeled anti-CD45 (30-F11, eBioscience), BV605-labeled anti-CD11b (M1/70, BioLegend). Fixable Viability Dye 780 (eBioscience) (1:1000) was added as a live/dead stain to the antibody mix. Cells were acquired on a BD LSR Fortessa SORP flow cytometer. Analysis was performed with FlowJo VX. Experiments were performed under corresponding tissue normoxic conditions.

#### Seahorse XF cell mitochondrial stress test

To determine the oxygen consumption rate (OCR) and the extracellular acidification rate (ECAR) of cultured TRM, Cell Mito Stress Tests (Agilent) were performed using a Seahorse XFe96 Analyzer (Agilent). One day before the assay, TRM-LCs were detached and counted using a Neubauer counting chamber. Either 70.000 cells (MG-LCs, KC-LCs, PM-LCs) or 35.000 cells (AM-LCs) were replated into a Seahorse XF96 Cell Culture Microplate (Agilent) in the corresponding medium. To provide experimental data on the bioenergetic profile of activated macrophages, the cells were either not stimulated to serve as a control or stimulated with either LPS (100 ng/mL), poly(I:C) (25 μg/mL) or zymosan (10 μg/mL) for 24 h. The sensor cartridge was rehydrated overnight with sterile H_2_O in an incubator at 37°C. On the day of the experiment, the sensor cartridge was rehydrated with overnight heated XF Calibrant (Agilent). Cells were washed and Seahorse base medium was added. Cells were incubated for 1 h at 37°C without CO_2_. OCR and ECAR were analyzed in response to 3 μM oligomycin (Sigma), 1 μM carbonyl cyanide-p-trifluoromethoxyphenylhydrazone (FCCP) (Sigma) and 2 μM antimycin A (Sigma) and 2,5 μM rotenone (Sigma). The Cell Mito Stress Test was performed according to manufacturer’s instructions with 3 min mixture time and 3 min measurement time for each of the 3 injections. Experiments were performed under corresponding tissue oxygen conditions including the oxygen specific basis Seahorse protocol. Data were analyzed using Seahorse Wave software (Agilent).

### Quantification and statistical analysis

No statistical methods were used to predetermine sample sizes. GraphPad Prism5 and 9 were used for the following statistical tests: Comparisons of gene expression, cytokine secretion and metabolic energy profile in Seahorse assays between more than two groups were made by one-way ANOVA with Tukey post-hoc test. Phagocytosis rate of different macrophages at different time points was compared using a two-way ANOVA with Bonferroni post-hoc test. Differences were considered statistically significant at p < 0.05. Data are presented as mean ± SEM, unless indicated otherwise. Statistical significance is indicated with asterisks: ∗p < 0.05, ∗∗p < 0.01 and ∗∗∗p < 0.001.

## Data Availability

• RNA-seq data have been deposited at Gene Expression Omnibus and are available under the ID GSE196376 using the token qnmraksexbgrrux. The data is publicly available as of the date of publication. Microscopy and flow cytometry data reported in this paper will be shared by the lead contact upon request. • No original code was generated in this study, all code used in this study is publicly available as outlined in the method details. • Any additional information required to reanalyze the data reported in this paper is available from the lead contact upon request.

## Supplemental information

Document S1. Figures S1–S7

Table S1. Differentially regulated genes of cultured TRM-LCs and *ex vivo* isolated TRMs of the respective organs, related to Figure 4 The first column indicates whether the genes are UP in *ex vivo*, UP in *in vitro*, or shared (NOT significantly regulated). Each cell type is presented and compared (*in vitro* versus *ex vivo*) in a separate sheet.

Document S2. Article plus supplemental information
