# Supplementary material for: An improved organotypic cell culture system to study tissue-resident macrophages ex vivo
Source: Cell Rep Methods. 2022 Jul 26;2(8):100260. doi: 10.1016/j.crmeth.2022.100260 (PMC9421540; doi:10.1016/j.crmeth.2022.100260)
Supplement: Document S1. Figures S1–S7 [file mmc1.pdf]

Cell Reports Methods, Volume *2*

Supplemental information

An improved organotypic cell culture system to study tissue-resident macrophages *ex vivo*

Philipp Aktories, Philippe Petry, Paulo Glatz, Geoffroy Andrieux, Alexander Oschwald, Hannah Botterer, Oliver Gorka, Daniel Erny, Melanie Boerries, Philipp Henneke, Olaf Groß, Marco Prinz, and Katrin Kierdorf

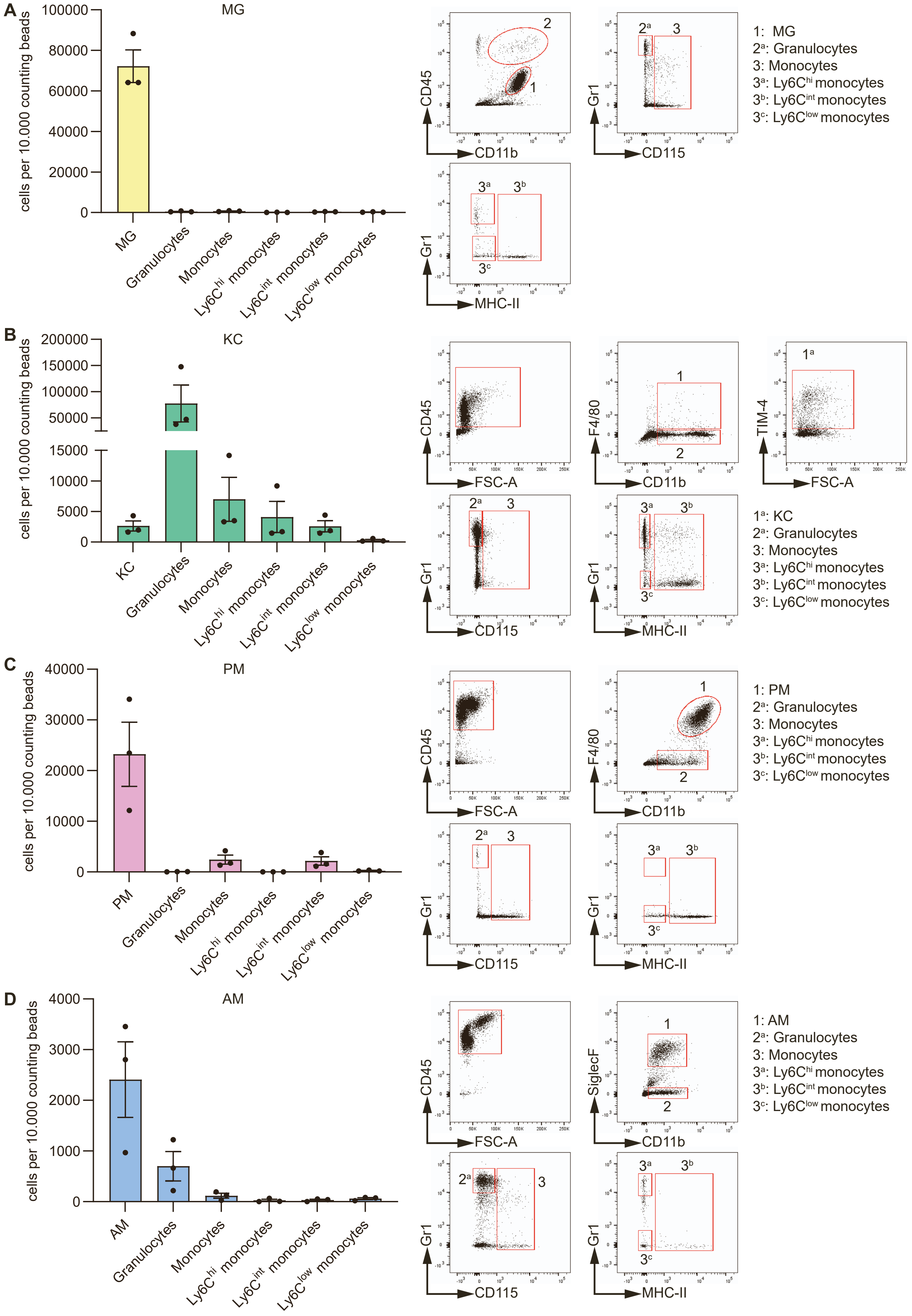

Suppl. Figure 1

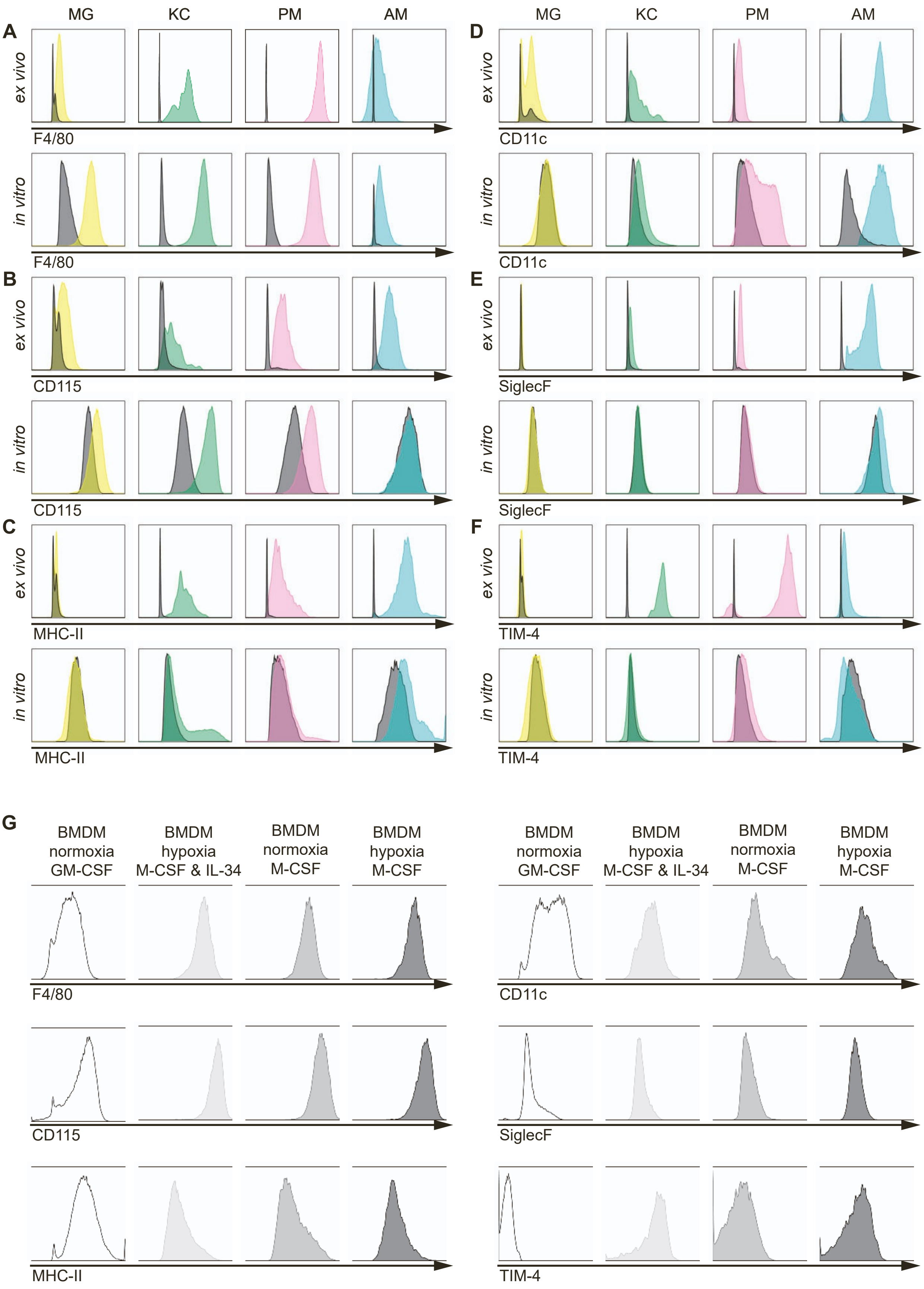

Suppl. Figure 2

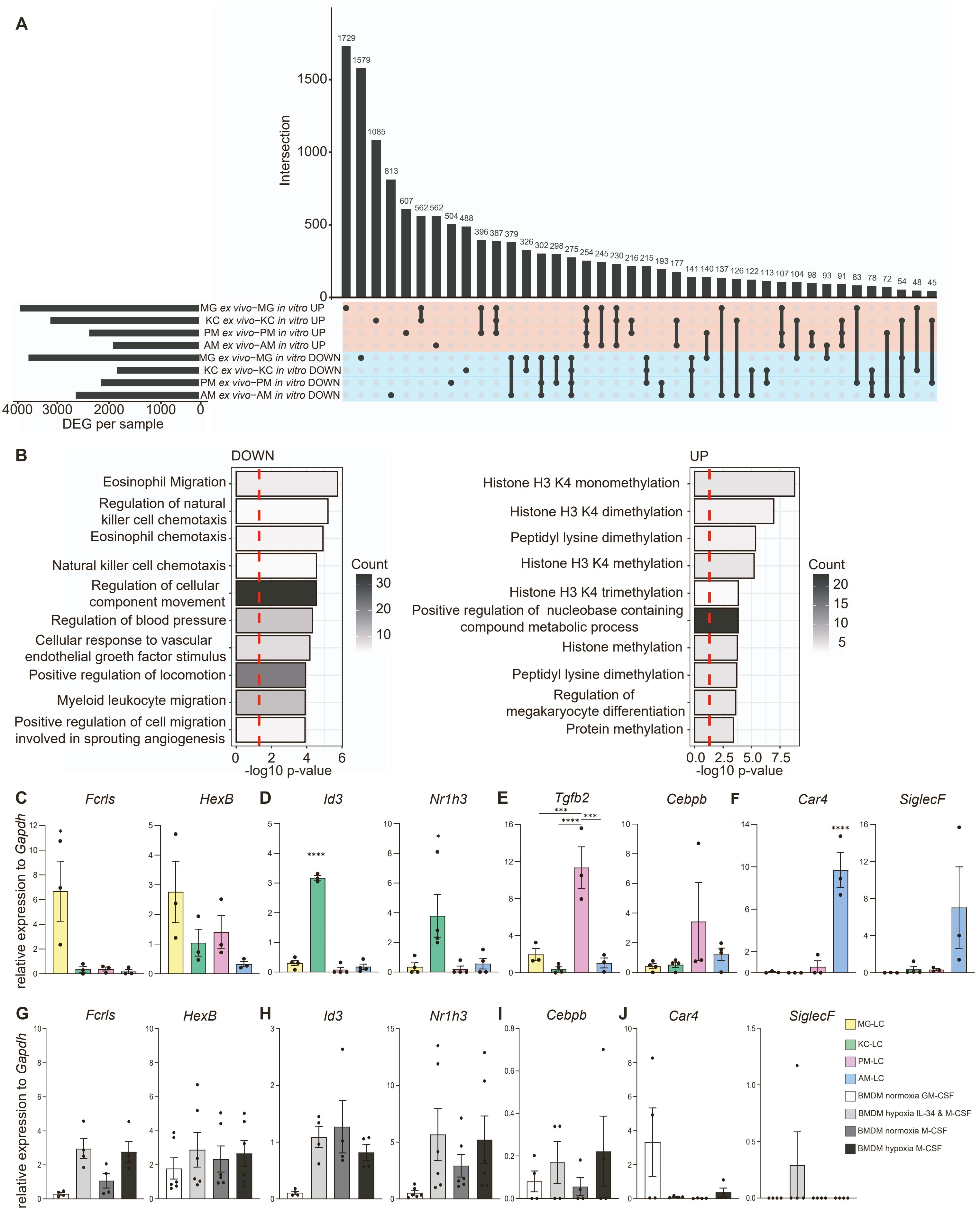

Suppl. Figure 3

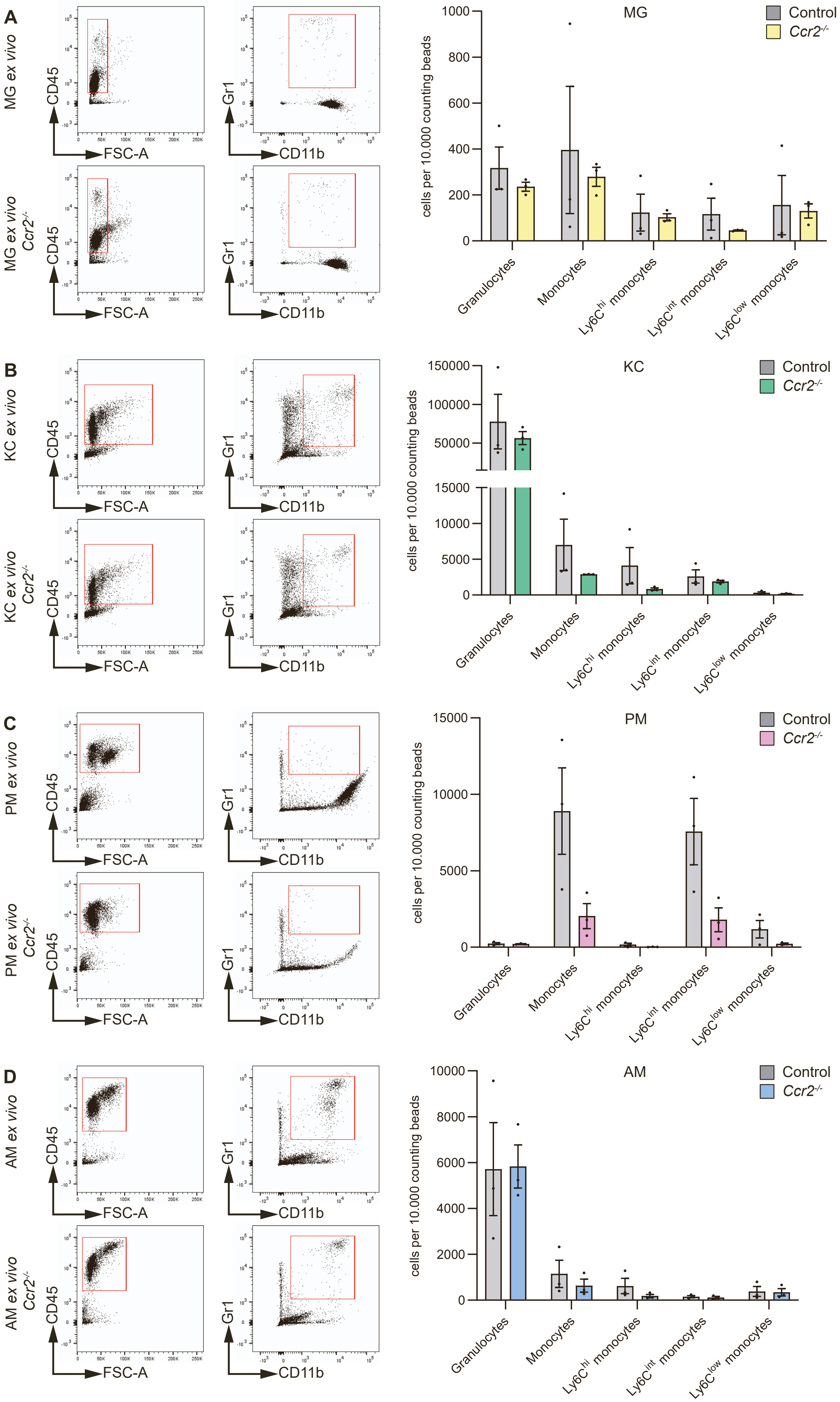

Suppl. Figure 4

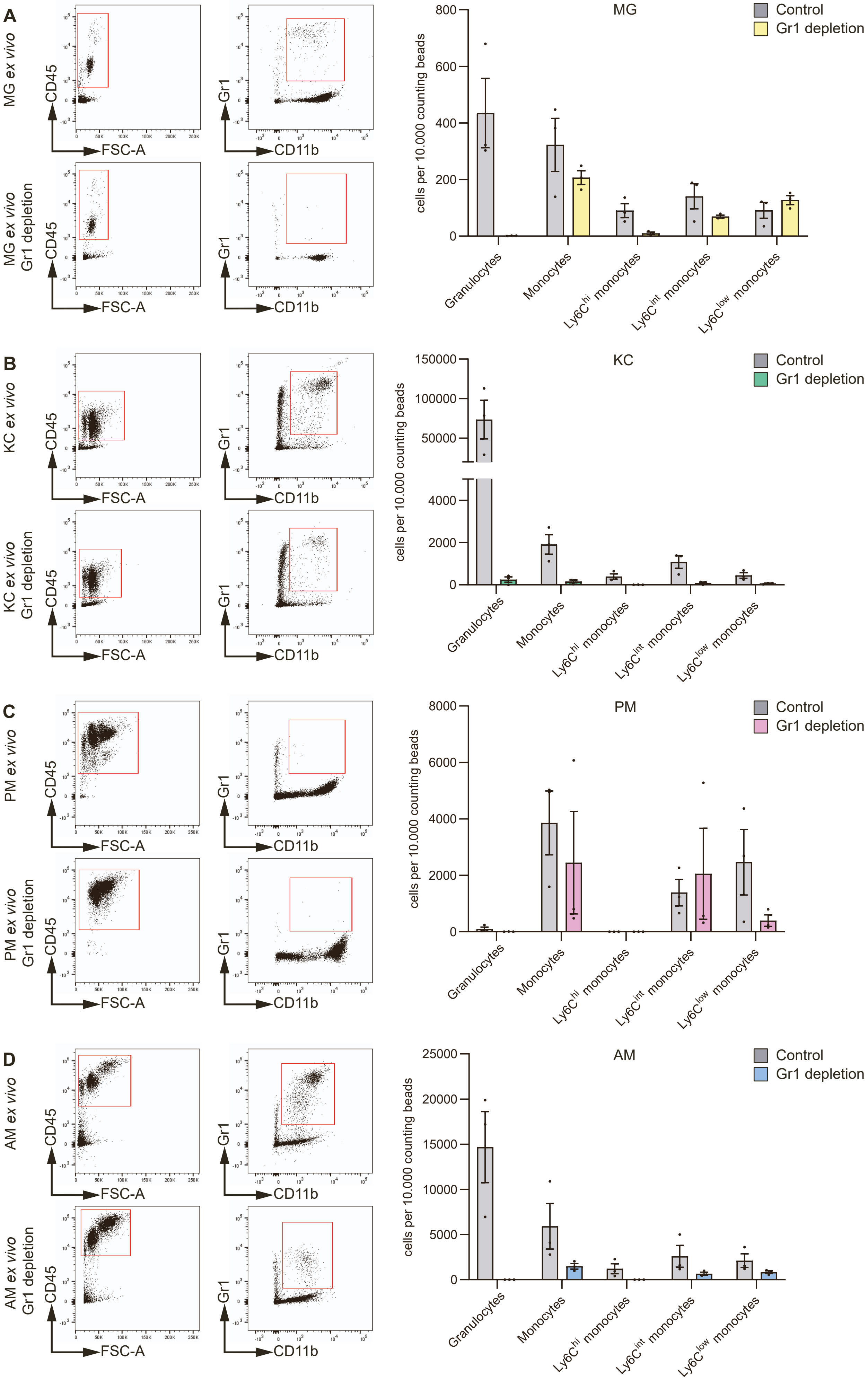

Suppl. Figure 5

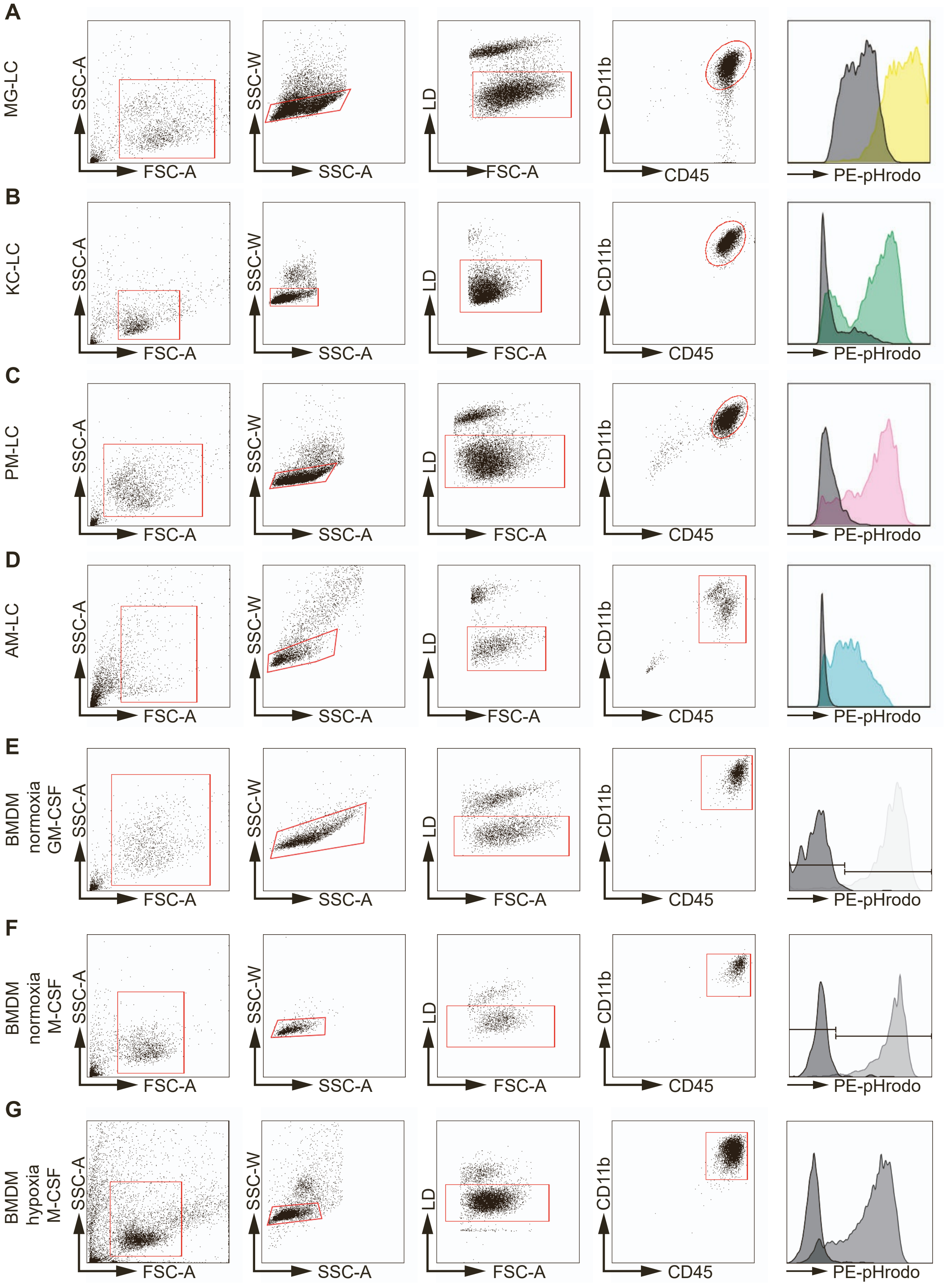

Suppl. Figure 6

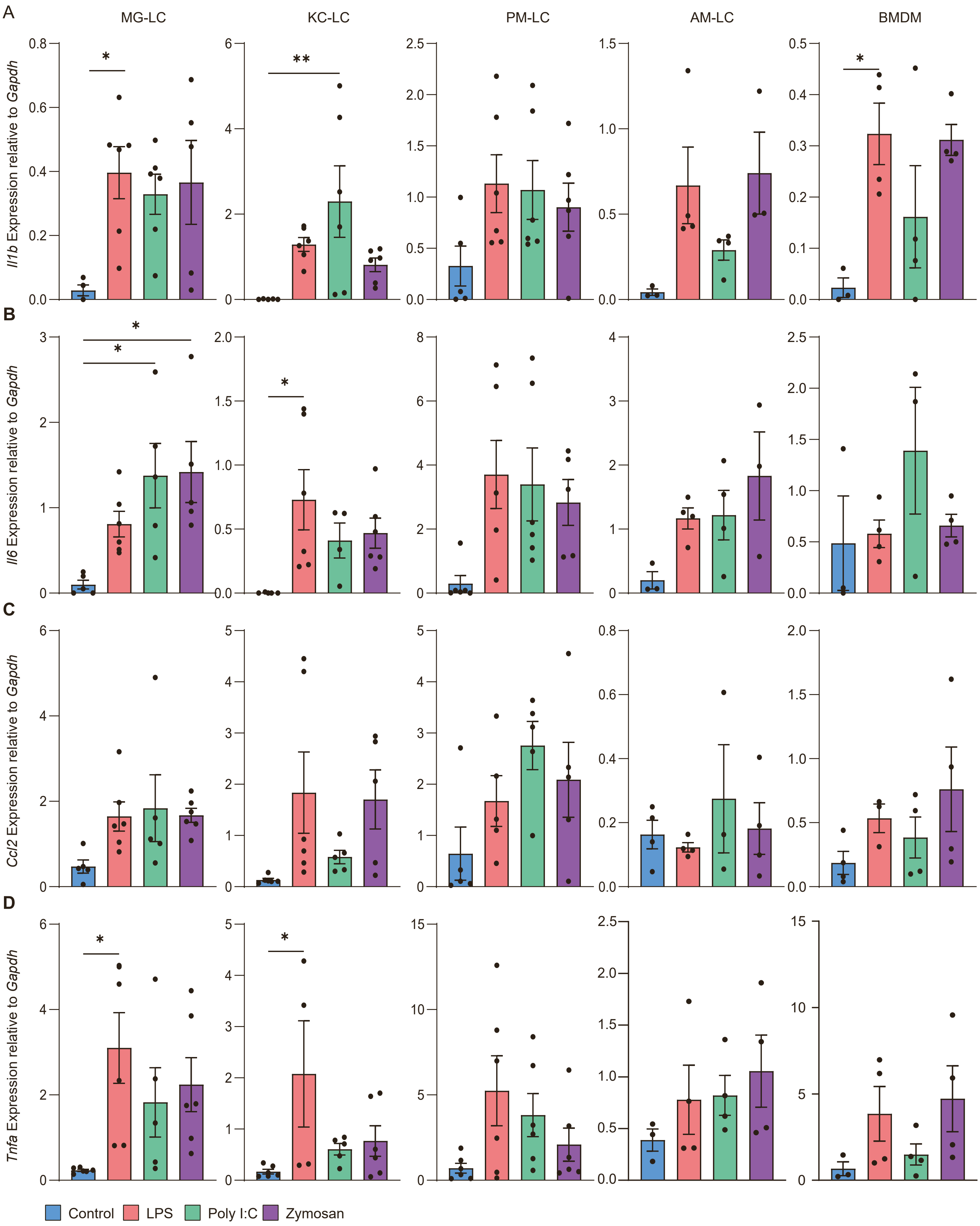

Suppl. Figure 7

Supplemental item titles and legends

Suppl. Figure 1: Isolated cells for plating contain a mixture of myeloid cells, related to Figure 1. Quantification of cell composition of cell pellets per 10.000 counting beads, n=3/organ. One out of three independent experiments is shown. Gating strategies are shown from left to right, gates are indicated in red (right side). Myeloid cell subsets are indicated with numbers. TRM are shown in gate $1^{(a)}$, granulocytes in gate 2^{a}, monocytes in gate 3, as well as $Ly6C^{hi}$ monocytes in gate 3^{a}, $Ly6C^{int}$ monocytes in gate 3^{b} and $Ly6C^{low}$ monocytes in gate 3^{c}. Quantification and gating strategy for cell pellets from brain (A), liver (B), peritoneum (C) and lung (D) are shown.

Suppl. Figure 2: Surface marker profiling of cultured TRM-LC compared to *ex vivo* isolated TRM, related to Figure 3. Macrophage subsets were identified as shown before in Figure 3. MG are shown in yellow, KC in green, PM in pink and AM in blue. Unstained controls are shown in grey. One out of 3 independent experiments is shown. (A-F) Histograms for *ex vivo* isolated macrophages (upper row) and *in vitro* cultured TRM-LC (lower row) are shown. Histograms are shown for F4/80 (A), CD115 (B), MHC-II (C), CD11c (D), SiglecF (E) and TIM-4 (F). (G) Histograms for cell surface markers on BMDM cultured in different culture conditions *in vitro*. One out of 3 independent experiments is shown. BMDM in normoxia + GM-CSF are shown in white, BMDM in hypoxia + M-CSF & IL-34 are shown in light grey, BMDM in normoxia + M-CSF are shown in grey, BMDM in hypoxia + M-CSF are shown in dark grey. Representative histograms are shown for F4/80 (upper left), CD11c (upper right), CD115 (middle left). SiglecF (middle right), MHC-II (lower left) and TIM-4 (lower right).

Suppl. Figure 3: TRM-LC do not share a transcriptomic *in vitro* signature maintain expression of specific TRM signature genes *in vitro*, related to Figure 4. (A) Intersection of genes significantly regulated (absolute log2 fold change > 1.5 and adjusted p-value < 0.05) in TRM-LC. (B) Enrichment analysis (Gene Ontology Biological Processes) of the 275 down-regulated genes shared between all TRM-LC (left panel) and the 254 up-regulated genes shared between all TRM-LC (right panel). (C-F) Gene expression analysis for signature genes in TRM-LC is shown. MG-LC are shown in yellow, KC-LC in green, PM-LC in pink, AM-LC in blue. Mean ± SEM is shown. n=3-4 per group. * $p<0.05$ and *** $p<0.001$. Gene expression is shown for *Hexb* (left) and *Fcrls* (right) (C), *Id3* (left) and *Nr1h3* (right) (D), *Cebpb* (left) and *Tgfb2* (right) (E), and *Car4* (left) and *Siglecf* (right) (F). (G-J) Gene expression analysis for signature genes in BMDM. BMDM in normoxia + GM-CSF are shown in white, BMDM in hypoxia + M-CSF & IL-34 are shown in light grey, BMDM in normoxia + M-CSF are shown in grey, BMDM in hypoxia + M-CSF are shown in dark grey. Mean ± SEM is shown. n=3-4 per group. Gene expression is shown for *Hexb* (left) and *Fcrls* (right) (G), *Id3* (left) and *Nr1h3* (right) (H), *Cebpb* (I), and *Car4* (left) and *Siglecf* (right) (J).

Suppl. Figure 4: $Ccr2^{-/-}$ mice have a reduced number of $Ly6C^{high}$ monocytes in the cell pellets used for TRM-LC cultures, related to Figure 5. Left: Representative flow cytometry of cell pellets from WT mice (control) and $Ccr2^{-/-}$ mice is shown prior to plating. Representative FACS plots are shown from left to right, gates are indicated in red. Full gating strategy is not shown. Doublets and dead cells were excluded before. Right: Relative quantification of myeloid cells within the plated cell pellets, normalized to counting beads. One out of three independent experiments are shown. Quantification is shown for MG (A), KC (B), PM (C), and AM (D).

Suppl. Figure 5: MACS-depletion of $Gr1^{+}$ cells is highly efficient, related to Figure 5. Left panels: Representative flow cytometry of undepleted (control) and $Gr1^{+}$ depleted cell pellets is shown prior to plating. Representative FACS plots are shown from left to right, gates are indicated in red. Full gating

strategy is not shown. Doublets and dead cells were excluded before. Right panels: Relative quantification of myeloid cells within the plated cell pellets, normalized to counting beads. One out of three independent experiments are shown. $Gr1^+$ cell depletion efficiency is shown for MG (A), KC (B), PM (C), and AM (D).

Suppl. Figure 6: Simplified gating strategy and Cytochalasin-D control for phagocytosis assay, related to Figure 6. Phagocytosis assays were performed with TRM after two weeks in culture. Gating strategy and PE-pHrodo histogram is depicted for MG-LC (A), KC-LC (B), PM-LC (C), AM-LC (D), and BMDM (hypoxia + M-CSF) (E). Representative histograms indicate PE-pHrodo labeling for the specific TRM-LC compared to the Cytochalasin-D control (grey) after 30 minutes. One out of 3-6 independent experiments is shown.

Suppl. Figure 7: TRM-LC show distinct inflammatory gene expression upon immune stimulation *in vitro*, related to Figure 6. Gene expression levels are shown relative to *Gapdh*. (A) *Il1b* expression in TRM-LC and BMDM. (B) *Il6* expression in TRM-LC and BMDM. (C) *Ccl2* expression in TRM-LC and BMDM. (D) *Tnfa* expression in TRM-LC and BMDM. Control is shown in blue, LPS treated group is shown in red, poly I:C treated group is shown in green, zymosan treated group is shown in violet. Mean ± SEM is shown. n=3-6 is shown. * $p<0.05$, ** $p<0.01$ and *** $p<0.001$
